# A Bayesian Meta-Analysis of Multiple Treatment Comparisons of Systemic Regimens for Advanced Pancreatic Cancer

**DOI:** 10.1371/journal.pone.0108749

**Published:** 2014-10-06

**Authors:** Kelvin Chan, Keya Shah, Kelly Lien, Doug Coyle, Henry Lam, Yoo-Joung Ko

**Affiliations:** 1 Sunnybrook Odette Cancer Centre, University of Toronto, Toronto, ON, Canada; 2 Division of Biostatistics, Dalla Lana School of Public Health, University of Toronto, Toronto, ON, Canada; 3 University of Ottawa, Ottawa, ON, Canada; Thomas Jefferson University, United States of America

## Abstract

**Background:**

For advanced pancreatic cancer, many regimens have been compared with gemcitabine (G) as the standard arm in randomized controlled trials. Few regimens have been directly compared with each other in randomized controlled trials and the relative efficacy and safety among them remains unclear.

**Methods:**

A systematic review was performed through MEDLINE, EMBASE, Cochrane Central Register of Controlled Trials, and ASCO meeting abstracts up to May 2013 to identify randomized controlled trials that included advanced pancreatic cancer comparing the following regimens: G, G+5-fluorouracil, G+ capecitabine, G+S1, G+ cisplatin, G+ oxaliplatin, G+ erlotinib, G+ nab-paclitaxel, and FOLFIRINOX. Overall survival and progression-free survival with 95% credible regions were extracted using the Parmar method. A Bayesian multiple treatment comparisons was performed to compare all regimens simultaneously.

**Results:**

Twenty-two studies were identified and 16 were included in the meta-analysis. Median overall survival, progression free survival, and response rates for G arms from all trials were similar, suggesting no significant clinical heterogeneity. For overall survival, the mixed treatment comparisons found that the probability that FOLFIRINOX was the best regimen was 83%, while it was 11% for G+ nab-paclitaxel and 3% for G+ S1 and G+ erlotinib, respectively. The overall survival hazard ratio for FOLFIRINOX versus G+ nab-paclitaxel was 0.79 [0.50–1.24], with no obvious difference in toxicities. The hazard ratios from direct pairwise comparisons were consistent with the mixed treatment comparisons results.

**Conclusions:**

FOLFIRINOX appeared to be the best regimen for advanced pancreatic cancer probabilistically, with a trend towards improvement in survival when compared with other regimens by indirect comparisons.

## Introduction

Pancreatic cancer is the 4^th^ leading cause of cancer death in the United States and 5^th^ in the United Kingdom [1], [2] with most cases being categorized as either metastatic or locally advanced at first presentation [3]. As potentially curative surgical resection can be performed in only 15–20% of pancreatic cancer patients [4], the treatment goal for the majority of these patients is palliative in nature. For more than 15 years, the current standard of care for advanced disease has been chemotherapy with gemcitabine alone (G), after it was shown in a phase III randomized control trial (RCT) to offer greater symptom relief with a modest 1-year survival advantage (18% versus 2%) when compared to 5- fluorouracil [5]. Since then, a number of phase II and III RCTs have attempted to improve the gemcitabine anti-tumour activity through gemcitabine-based combinations with cytotoxic and/or targeted agents such as capecitabine, oxaliplatin, erlotinib, and cisplatin [6]–[10]. Recent trials have also compared gemcitabine alone to gemcitabine plus nab-paclitaxel (GnP), and a combination regimen without gemcitabine consisting of folinic acid, fluorouracil, irinotecan hydrochloride and oxaliplatin (FOLFIRINOX) [11], [12]. The trial of G versus GnP found statistically significant hazard ratios (HRs) for overall survival (OS) in favour of the GnP combination. The safety analysis found that serious life-threatening toxicity was not increased with GnP and that adverse events were acceptable and manageable. Thus, the authors concluded that GnP may be considered as a new standard of treatment for advanced pancreatic cancer [11]. In the FOLFIRINOX trial, survival was significantly better in the FOLFIRINOX group, but with an increased occurrence of adverse events. The study concluded that FOLFIRINOX should also be considered as a first-line option for advanced pancreatic cancer patients; however, due to safety concerns, it should be reserved for patients younger than 75 years of age and with a good performance status [12]. No currently ongoing trials directly compare GnP and FOLFIRINOX. While the addition of these two chemotherapy regimens and their improvement in survival represent significant recent progress over gemcitabine monotherapy, the most effective chemotherapy strategy in clinical practice remains to be determined.

As direct comparison of combination therapies has been tested mostly against single agent gemcitabine as the control arm in most clinical trials, the relative effectiveness of the various regimens remains unclear. In these instances, multiple treatment comparisons (MTC) can be used to synthesize evidence from RCTs using both direct (head-to-head) and indirect (using a common comparator) comparisons [13]. MTC are valuable tools that are frequently employed by healthcare decision makers such as the National Institute for Health and Clinical Excellence and the Canadian Agency for Drugs and Technologies in Health, where their usage is gaining widespread acceptance [14], [15].

The aim of this study was to perform Bayesian MTC in order to determine the most effective treatment for advanced pancreatic cancer, taking into account the efficacy and safety profiles of each regimen. Through our analysis, we were able to achieve this goal.

## Methods

### Literature Search

We conducted a systematic literature review through the MEDLINE, EMBASE, and Cochrane Centre Register of Controlled Trials databases, as well as ASCO meeting abstracts up to and including May 23, 2013. Trials were limited to first-line treatment in pancreatic cancer or adenocarcinoma patients. Studies were limited to randomized controlled trials (RCTs) that used one of the following chemotherapy regimens: G, G + fluorouracil (GF), G + capecitabine (GCap), G + S1 (GS), G + cisplatin (GCis), G + oxaliplatin (GOx), G + erlotinib (GE), GnP, and FOLFIRINOX. These regimens were determined a priori by the authors, as they are clinically the most commonly considered treatments for advanced pancreatic cancer with prior studies suggesting possible benefits to patients. The outcomes of interest included OS, progression-free survival (PFS), and grade 3/4 toxicities. RCTs that did not include patients with advanced pancreatic cancer were excluded. Non-randomized trials and those concerning other malignancies, such as neuroendocrine tumours or lymphoma, were excluded. Trials comparing radiotherapy, hormonal, or gene therapy, and those comparing chemotherapy to no treatment (best supportive care) were excluded. No language restrictions were imposed. The articles that were not freely available to us were requested from the authors.

### Screening

Two independent authors reviewed the literature search results and included studies that met the prespecified eligibility criteria. When reports overlapped or were duplicated, we retained the study with the most recent data that could be used in the meta-analysis. Discrepancies were resolved by consensus or by a third author. Our review has been reported using the PRISMA reporting guidelines (Checklist S1).

### Data Abstraction and Analysis

Data recorded included the following: first author, publication year, study location, regimens being compared, number of patients randomized to each treatment arm, median age of patients, percentage of patients with performance status of ECOG 0, 1, or 2 and the percentage of patients with locally advanced or advanced disease respectively was recorded (Appendix S1 and S2). The treatments were sorted into categories based on the regimen: G, GF, GCap, GS, GCis, GOx, GE, GnP, and FOLFIRINOX. Risk of bias assessment was performed using the Cochrane risk of bias tool [16].

The data extracted from each study included the following: OS, PFS, objective response rate (ObRR), and the occurrence of adverse events (febrile neutropenia, neuropathy, fatigue, and diarrhea) for all the chemotherapy regimens. If median values for PFS and OS were available, they were also recorded. If the HRs for OS and PFS were detailed in the publication, they were extracted directly, along with 95% confidence intervals (CIs) from Cox regression. Otherwise, HRs were calculated using the methods outlined by Parmar et al [17]. A two-tailed p<0.05 value was recorded whenever available to determine whether a statistically significant difference was detected between the two regimens being compared. Two independent authors extracted data and discrepancies were reviewed by a third author to reach consensus.

### Statistical Analysis

We first made pairwise comparisons of regimens from the trials based on direct evidence only. We then performed MTC in a Bayesian model. The MTC combined direct and indirect evidence for specific pairwise comparisons and allowed data across a range of regimens to be compared in a simple network. Bayesian methods combine likelihoods, as a function of the parameters with a prior probability distribution based on previous knowledge, to obtain a posterior probability distribution of the parameters [18]. The posterior probabilities provide a straightforward way to calculate the most effective treatment in the absence of head-to-head trials. By plotting the posterior densities of the direct, indirect, and network estimates, direct and indirect evidence can be combined to provide a network estimate and a single effect size. This effect size has increased precision than that of any one type of evidence alone. The Bayesian approach has undergone significant development in recent years and is able to monitor convergence in posterior distribution and reflect the uncertainty in estimating heterogeneity, offering significant improvements over the frequentist random-effects model, which cannot estimate that uncertainty. In more complex networks, especially those involving multi-armed trials, Bayesian approaches are more developed and more accessible than their frequentist counterparts [18], [19].

Analyses were done using Bayesian Markov Chain Monte-Carlo (MCMC) sampling in WinBUGS, version 1.4.3 and reported according to the Quality of Reporting of Meta-analyses (QUOROM) and International Society for Pharmacoeconomics and Outcomes Research (ISPOR) guidelines. In WinBUGS, 3 chains were fit with 40,000 burn-ins and 40,000 iterations each. Assessment of convergence was done using model diagnostics, such as trace plots and the Brooks-Gelman-Rubin statistic [20]. Model fit was determined based on the residual deviance and deviance information criterion (DIC) for each outcome measure. The random effects model was used for OS, PFS, and ObRR because the residual deviance was less than the number of unconstrained data points and the deviance information criterion for each of these outcome measures favoured this model over the fixed effects model. Fixed effects were used in reporting toxicities because the residual deviance and DIC favoured this model. We used the following non-informative prior distributions: uniform (0,2) for standard deviation of the random effects model and normal (0, tau  = 0.0001) for log[HR]s. Non-informative priors were used because this allowed the trial data to inform the results, rather than letting strong priors dictate the results.

The primary endpoint was OS and the secondary endpoints were PFS and ObRR. OS and PFS were summarized as log[HR], ObRR and toxicities were summarized as log[Odds Ratio]. Effect sizes are described with 95% credible regions (CRs), since “credible” is a more appropriate term than “confidence” when conducting Bayesian MTC. Consistency between direct and indirect evidence was assessed by comparing direct pairwise comparison estimates to the results generated in the MTC. Probability of each regimen being the best among all regimens were computed by ranking the relative efficacies of all regimens in each iteration and then calculating the proportion of each regimen being ranked first across all iterations [21]. In order to assess the comparability of included studies, between-study heterogeneity was estimated and reported using the I^2^ statistic; the value of I^2^ lies between 0% and 100%, where 0% indicates no observed heterogeneity and larger values show increasing heterogeneity [17].

Based on the HR results of the MTC, we attempted to project the survival of patients receiving each of the regimens and compared the results to the median OS of G. Projected median OS was calculated using a median OS of 5.65 months for G as reported by Buris et al [5]. Survival was estimated based on the MTC results and the methods presented by Altman and Andersen [22].

## Results

### Literature Search Results

**Figure 1:**
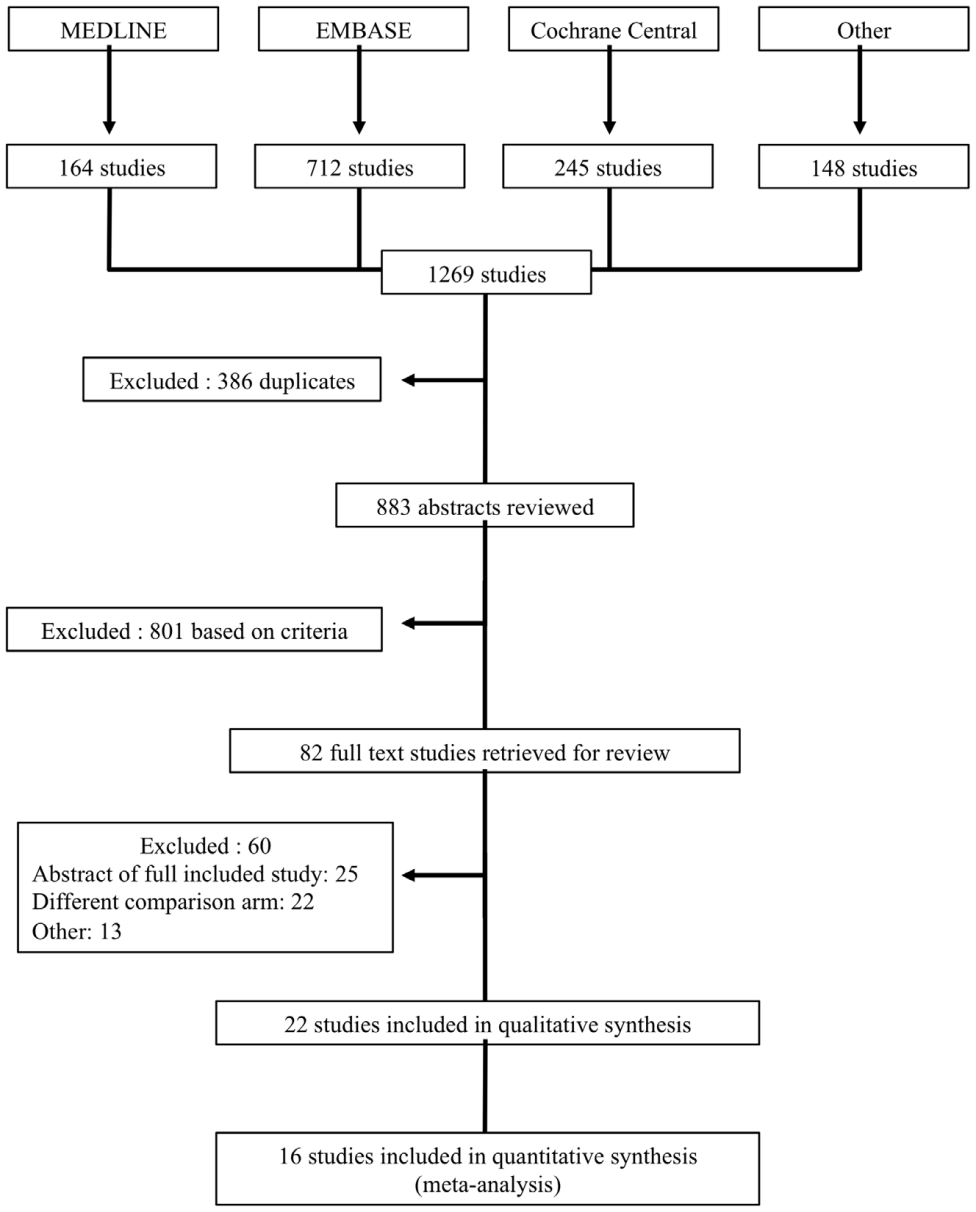
PRISMA Flow diagram of included and excluded trials identified from the literature search. There were 13 studies that were excluded after full text review for “other” reasons. The reasons are as follows: 4 were secondary analyses, 2 were quality of life studies, 2 were pooled analyses, 1 study was not randomized, 1 was a review, 1 was a tumour marker study, 1 was a safety analysis, and 1 study was excluded because it was retrospective.

Figure 1 shows a flow diagram of the selection process for the studies included in our meta-analysis. 1269 studies were identified from the literature search, 386 studies were excluded because they were duplicates, and 801 were excluded after the abstracts were reviewed based on the prespecified criteria. Of the 82 studies that underwent full text review, 25 were excluded because they were an abstract of a full-included study, 22 had a different comparison arm, 4 were secondary analyses, 2 were quality of life studies, 2 were pooled analyses, 1 study was not randomized, 1 was a review, 1 was a tumour marker study, 1 was a safety analysis, and 1 study was excluded because it was retrospective. Twenty-two studies were identified to be included in this review [6]–[12], [23]–[38]. 16 studies, involving 5488 randomized patients contained sufficient data to be included in the quantitative synthesis (meta-analysis). The studies included in the meta-analysis consisted of 15 manuscripts and 1 ASCO meeting abstract, which was subsequently published as a full manuscript [38]. The subsequent publication was reviewed and the results were verified and found to be identical to the results reported in the original abstract [11], [38].

### Study Quality

A summary of the risk of bias for each included study can be found in Appendices S14 and S15. All included studies were randomized and 12 out of the 16 studies followed intention-to-treat analysis for the primary endpoint, thus minimizing selection bias and attrition bias, respectively. Only one study had blinding of patients or personnel. Although blinding of outcome assessors was not explicitly indicated, 13 studies had OS as the primary endpoint, which would not be influenced by the outcome assessor. Therefore there is a low risk of detection bias in these studies. Allocation concealment was not mentioned in any of the studies, so some potential selection bias may be present.

### Trial Characteristics

**Figure 2:**
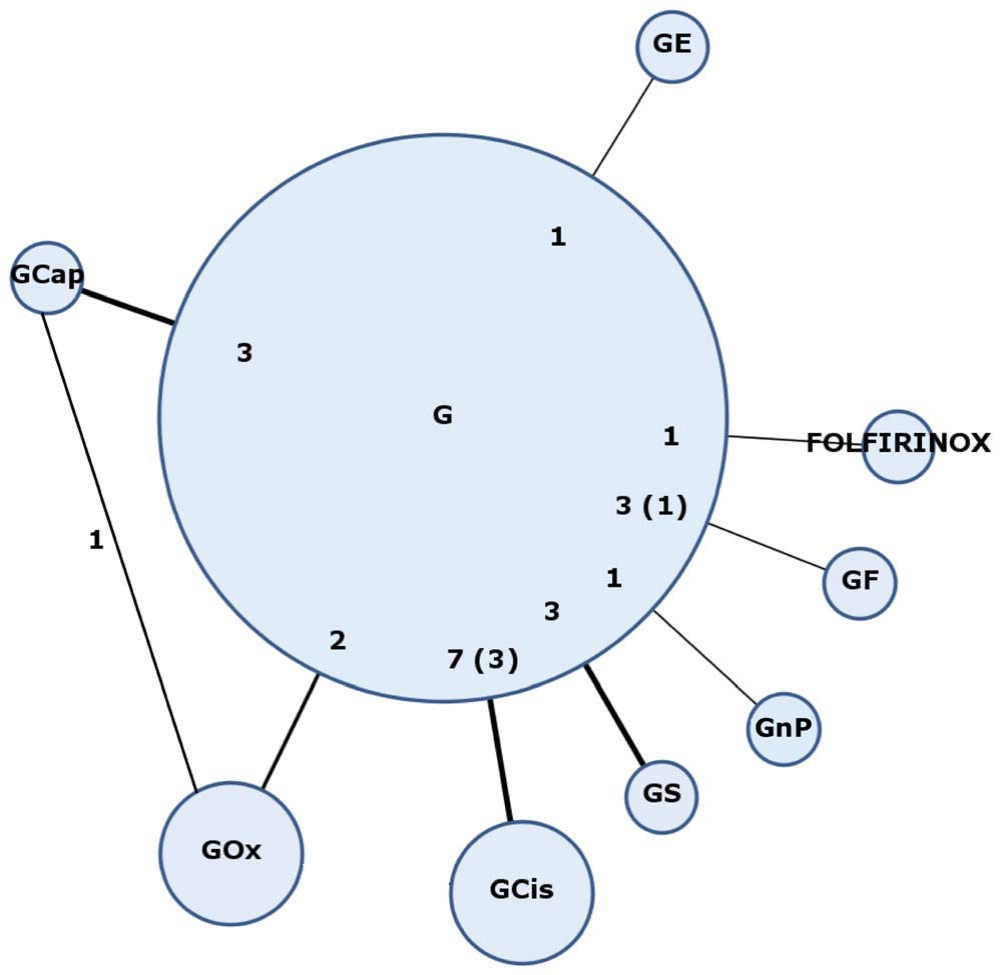
Treatment strategy network. Numbers represent the number of studies comparing the linked regimens; brackets represent the number included in the quantitative analysis.

The chemotherapy regimens used in the included studies were G vs. GF (three studies), G vs. GCap (three studies), G vs. GS (three studies), G vs. GCis (seven studies), G vs. GOx (two studies), G vs. GE (one study), G vs. FOLFIRINOX (one study), GCap + GOx (one study), and G + GnP (one study). The treatment strategy network is shown in Figure 2. All trials included in the meta-analysis reported median PFS and OS. There was no significant clinical heterogeneity between the studies based on the patient characteristics and outcomes in the G reference arm (median PFS  = 3 to 4 months, median OS  = 6 to 7 months) (Appendix S3).

### Comparison of Regimens

**Figure 3:**
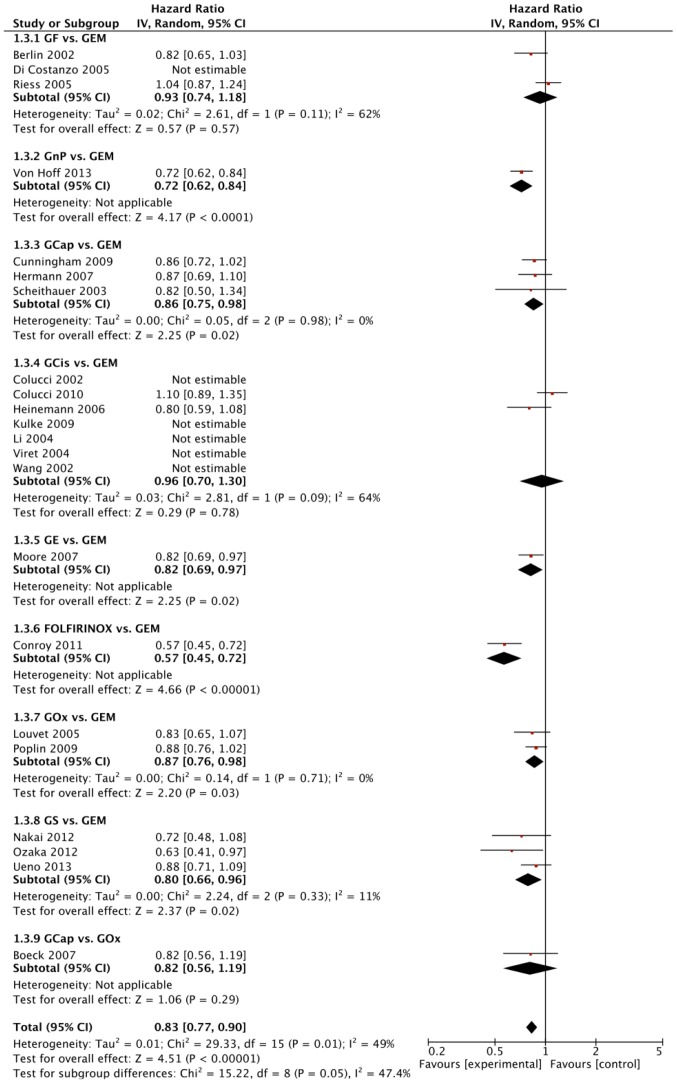
Forest plot of direct comparisons between the regimens. Forest plot showing hazard ratio comparisons with 95% CI for overall survival (OS) from meta-analyses of direct comparisons between different combinations of gemcitabine (GEM), gemcitabine + fluorouracil (GF), gemcitabine + nab-paclitaxel (GnP), gemcitabine + capecitabine (GCap), gemcitabine + cisplatin (GCis), gemcitabine + erlotinib (GE), FOLFIRINOX, gemcitabine + oxaliplatin (GOx), and G + S1 (GS). I^2^ values indicate statistical heterogeneity, where 0% indicates no observed heterogeneity and larger values show increasing heterogeneity (17).

The outcomes assessed in all the trials were OS, PFS, ObRR, and number of toxicity-related adverse events. Of the 16 trials that compared different regimens, seven found statistically significant differences in OS based on direct evidence only (Figure 3). These seven studies compared G alone to a different treatment arm. Direct comparisons detected statistically significant improvements in OS with GnP versus G (HR  = 0.72, [95% CR 0.62–0.84]), GCap versus G (HR  = 0.86, [0.75–0.98]), GE versus G (HR  = 0.82, [0.69–0.97]), FOLFIRINOX versus G (HR  = 0.57, [0.45–0.72]), GOx versus G (HR  = 0.87, [0.76–0.98]), and GS versus G (HR  = 0.80, [0.66 to 0.96]). These results can be seen in Figure 3. Statistical heterogeneity (I^2^>35%) was found only for the comparisons of GCis versus G (seven studies, I^2^ = 64%) and GF versus G (three studies, I^2^ = 62%) for OS. The direct comparisons for PFS with I^2^ values are shown in Appendix S4.

**Figure 4:**
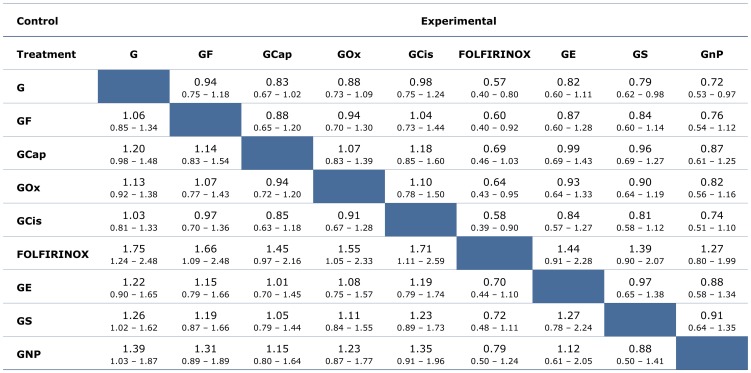
Hazard ratio comparisons of overall survival (OS) from mixed treatment comparisons. Median values given with 95% credible regions. Hazard ratios (HRs) expressed as experimental vs. control. G, gemcitabine; GF, gemcitabine + fluorouracil; GCap, gemcitabine + capecitabine; GOx, gemcitabine + oxaliplatin; GCis, gemcitabine + cisplatin; FOLFIRINOX; GE, gemcitabine + erlotinib; GS, gemcitabine + S1; GnP, gemcitabine + nab-paclitaxel.

**Figure 5:**
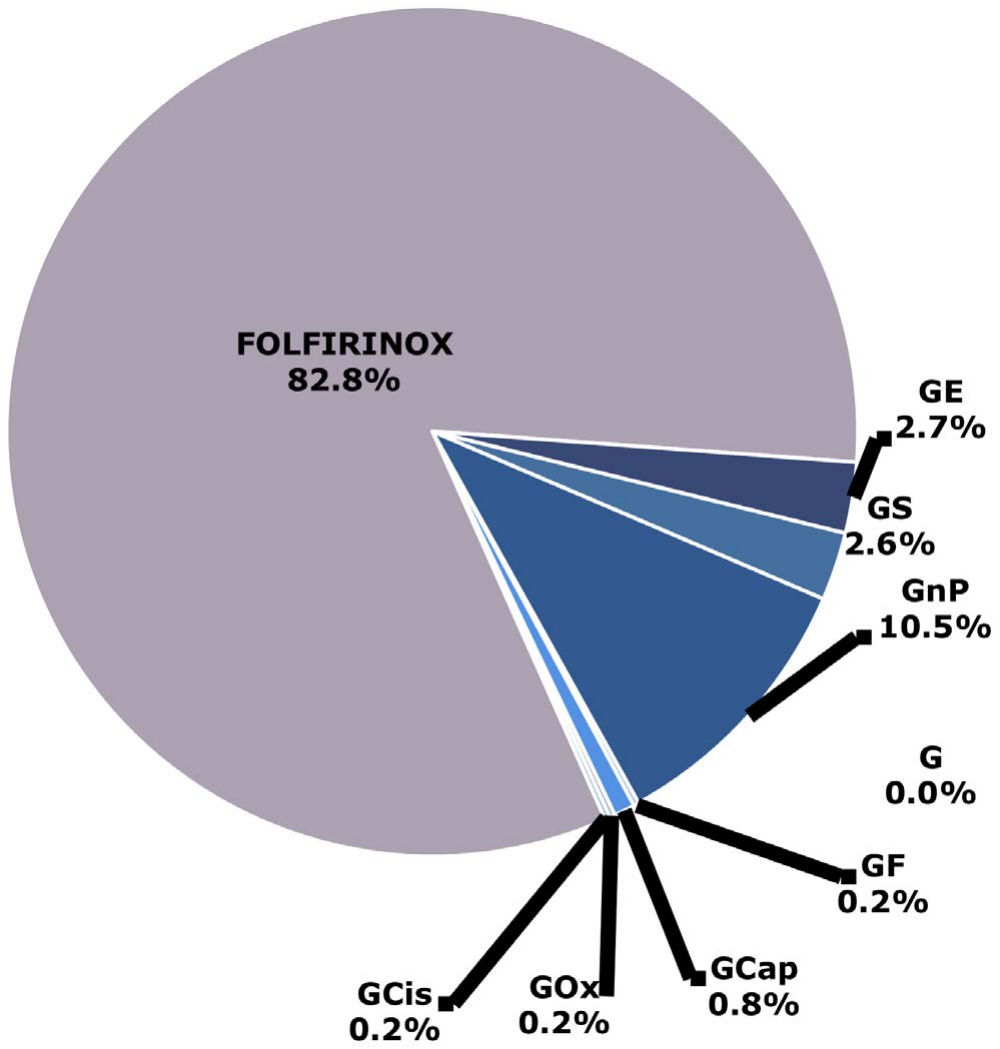
Probabilities that each treatment regimen is the best based on overall survival (OS). G, gemcitabine; GF, gemcitabine + fluorouracil; GCap, gemcitabine + capecitabine; GOx, gemcitabine + oxaliplatin; GCis, gemcitabine + cisplatin; FOLFIRINOX; GE, gemcitabine + erlotinib; GS, gemcitabine + S1; GnP, gemcitabine + nab-paclitaxel.

Through our Bayesian MTC, HR comparisons were made of OS (Figure 4) and PFS (Appendix S5) to compare all the regimens simultaneously. The results of the MTC were similar to the results seen in direct pairwise comparisons (Appendix S9). For OS, the results of the Bayesian MTC found that the probability that FOLFIRINOX was the best regimen was 83%, while it was 11% for GnP and 3% for GS and GE, respectively. For PFS, the Bayesian MTC found an 80% probability that FOLFIRINOX was the best regimen. Figure 5 shows the probabilities of each treatment regimen being the best in terms of OS. The probabilities for PFS can be seen in Appendix S6.

The next best regimens according to the calculated probabilities are GnP, GE, and GS. The OS HR for FOLFIRINOX versus GS was 0.72 [0.48–1.11], FOLFIRINOX versus GnP was 0.79 [0.50–1.24], and FOLFIRINOX versus GE was 0.70 [0.44–1.10], where HRs are given with 95% CRs. The PFS HR for FOLFIRINOX versus GS was 0.78 [0.47–1.40], FOLFIRINOX versus GnP was 0.68 [0.37–1.27], and FOLFIRINOX versus GE was 0.61 [0.33–1.15].

Projected survivals were estimated comparing each regimen to G. The projected median OS ranged from 5.8 months for GCis and 9.9 months for FOLFIRINOX (see Table 1). The number needed to treat (NNT) at 6 months and 1 year relative to G have been shown in Table 1. The NNT at 1 year ranges from 5 for FOLFIRINOX to 146 for GCis. These estimates will be helpful in clinical decision-making and providing information to patients.

**Table 1:** Comparisons of each regimen with Gemcitabine (G).

Regimen Name	OS Hazard Ratio when compared with G	Projected Median OS (months)*	NNT at 6 months when compared with G	NNT at 1 year when compared with G
FOLFIRINOX	0.57	9.9	6	5
G + nab-paclitaxel	0.72	7.8	9	9
G + S1	0.79	7.2	12	12
G + erlotinib	0.82	6.9	15	14
G + capecitabine	0.83	6.8	16	15
G + oxaliplatin	0.88	6.4	23	23
G + fluorouracil	0.94	6.0	46	47
G + cisplatin	0.98	5.8	141	146
G	—	5.65	—	—

Footnotes: Hazard ratios when comparing each regimen with Gemcitabine (G), projected median overall survival (OS), number needed to treat (NNT) at 6 months and 1 year when compared with G. Projected median OS was calculated using a median OS of 5.65 months as reported by Buris et al (5). Survival and NNT was estimated based on the mixed treatment comparisons results and the method by Altman and Andersen (22).

Odds ratio (OR) comparisons were made of ObRR (Appendix S7) to compare all the regimens simultaneously. The Bayesian MTC found a 58% probability that FOLFIRINOX is the best regimen in terms of ObRR, while it was 33% and 8% for GnP and GS respectively. The ObRR HR [95% CR] for FOLFIRINOX versus GnP is 1.59 [0.74–2.94]. The probabilities that each treatment regimen is the best in terms of ObRR are shown in Appendix S8.

The toxicity-related adverse events assessed in this study were febrile neutropenia and grade 3/4 fatigue, neuropathy, and diarrhea, as these are the most clinically relevant treatment related toxicities. ORs with 95% CRs were reported for each comparison with sufficient direct evidence available to make network estimates (Appendices S10, S11, S12, and S13). Based on cross-trial comparisons, there was no obvious difference in toxicities for FOLFIRINOX and GnP. The raw numbers of toxicities from each included study can be found in Appendix S3.

When comparing the direct pairwise comparisons to the results generated from the MTC, we found that the results are consistent (Appendix S9).

## Discussion

### Key Findings and Implications

Based on the analysis of both the direct evidence and MTC, FOLFIRINOX had the highest probability of being the best regimen in terms of both OS (83%) and PFS (80%). In our study, selected comparisons of FOLFIRINOX with the regimens that had the next highest probabilities were also conducted. These results provide further evidence, albeit indirect, that FOLFIRINOX may be the most effective regimen in the treatment of advanced pancreatic cancer. Although this meta-analysis allows for network comparisons of FOLFIRINOX with other chemotherapy regimens, further large prospective trials with FOLFIRINOX and the other regimens, especially GnP, would ideally be performed to confirm these results.

For over the past 15 years, gemcitabine monotherapy has been the standard of care in many countries for the treatment of metastatic pancreatic cancer based on its modest clinical efficacy. Although the tumor response rate and survival benefit of gemcitabine is modest, its favorable toxicity profile and ease of administration has led to its wide spread and continued use. Many studies have attempted to improve on the efficacy of gemcitabine by adding either another chemotherapeutic agent or a targeted agent. However, the vast majority of the phase III studies conducted in this setting have been remarkably negative with the exception of the addition of erlotinib and more recently, nab-paclitaxel [38], [39]. Although the gemcitabine and erlotinib study demonstrated a statistically significant overall survival benefit in favour of the combination, the modest improvement in survival and higher toxicity likely influenced a more broad adoption of this regimen.

In addition, a population-based study conducted in 2012 examined the tolerance and effectiveness of FOLFIRINOX at three institutions [40]. The median PFS and OS reported in this study were 7.5 and 13.5 months respectively [40]. The PFS and OS from this study were actually higher than those from the pivotal randomized trial by Conroy et al [12]. However, this may be attributed to the fact that the population-based study included patients with all stages of pancreatic cancer, while the Conroy study enrolled only those with metastatic disease [12], [40]. With respect to adverse events, the observed rate of febrile neutropenia in the population-based study was 4.9%, which is similar to the rate observed in the Conroy study (5.4%), which suggests that the results of the clinical trial may be generalizable to an uncontrolled setting. This population-based study concluded that FOLFIRINOX was clinically effective in the treatment of advanced pancreatic adenocarcinoma and that the toxicity profile of the regimen does not outweigh the benefits in terms of ObRR and survival [12], [40]. Although FOLFIRINOX demonstrates the best overall survival, progression-free survival, and objective response rate as per the large Phase III Trial [12], it is important to note that this regimen has a higher toxicity profile. When comparing the safety profiles of FOLFIRINOX and GnP from two separate clinical trials, the rate of febrile neutropenia in patients treated with FOLFIRINOX was 5.4% [12], while it was 3% in the GnP group [11]. G-CSF was administered in 42.5% of patients receiving FOLFIRINOX [11] and in 26% of patients receiving GnP [12]. In addition, it is important to note that the FOLFIRINOX study excluded patients older than 75 years of age and those with an ECOG performance status of 2. Therefore, FOLFIRINOX may be more challenging to prescribe in elderly or frail patients and caution should be taken in these cases. Ongoing prospective population-based studies are being performed to assess the efficacy and safety of FOLFIRINOX outside of clinical trials, which will provide further real life experience of the regimen. In addition, no population-based studies conducted to evaluate the survival benefit and toxicity of GnP so further research should be done in order to compare FOLFIRINOX with GnP in clinical practice.

### Strengths and Limitations

There are a number of strengths of the current MTC. For example, a comprehensive and robust search strategy was used, with data being extracted by two authors independently to ensure accuracy. Although MTC allow indirect comparisons to be made, these indirect estimates may be influenced by potential biases and uncertainties. Multiple-treatment comparison meta-analysis should be interpreted with caution and specifically, the underlying assumptions of homogeneity and consistency of studies across the network should be carefully scrutinized. In our study, heterogeneity between studies was indeed assessed and reported using I^2^ values. Although some heterogeneity was noted in the comparisons of GCis versus G and GF versus G, all studies in included in the meta-analysis were comparable in terms of patient characteristics and outcomes in the G reference arm (median PFS  = 3–4 months, median OS  = 6–7 months). The HRs from direct pairwise comparisons and the MTC were also compared and found to be consistent (Appendix S9). A limitation of our analysis was the small number of studies included which is a reflection of the landscape of the medical evidence. For many of the comparisons, data was extracted from only one trial so any biases or limitations from that study were more likely to affect the conclusions drawn from the MTC. Another limitation of this method is that it is based on published group data, rather than individual patient information. Individual patient data may allow for more patterns to be seen in terms of risk factors, however, it would still remain difficult to make strong inferences in such a complex network of treatments.

Both the FOLFIRINOX and nab-paclitaxel trials included only those patients with metastatic pancreatic cancer in contrast to the other gemcitabine combination studies, which enrolled both metastatic and locally advanced pancreatic patients. One of the reasons behind this shift in patient profile of advanced pancreatic studies were the recommendations of a group of experts convened in 2009 by the National Cancer Institute in the United States based on the well described differences in survival between those with locally advanced and metastatic disease. Unfortunately, this difference in patient population across the trials included in our study could not be accounted for. However, given that the inclusion of locally advanced patients tends to magnify the overall and progression free survival, we do not expect this difference in the patients included in the studies to significantly influence our observed results.

As RCTs directly comparing FOLFIRINOX and GnP, or other existing regimens are unlikely to be conducted in advanced pancreatic cancer in the future due to both commercial and scientific reasons, indirect comparisons such as ours may represent the best possible level of evidence as to which regimen is best. Such indirect evidence may still in fact be informative in terms of both clinical and policy decision-making.

## Conclusions

Our meta-analysis reviewed and analyzed the existing high-quality evidence for treating advanced pancreatic cancer in an MTC, which help synthesize evidence and may inform decision-making in the absence of direct pairwise comparisons. Based on our MTC, FOLFIRINOX appears to be the most effective regimen, however, direct pairwise comparisons are warranted to definitively address. Existing uncertainties of the relative effectiveness of FOLFIRINOX, as well as the potential toxicities and long-term effects suggest that further clinical trials and longitudinal studies are needed.

## Supporting Information

Appendix S1 **Summary table of trial characteristics included in systematic review and quantitative synthesis.**
(TIFF)

Appendix S2 **Studies identified through the literature search.** The geographic location of the institution of the primary investigator is described in the case where no study location was specified.
(DOCX)

Appendix S3 **Extracted data for PFS, OS, ObRR, and side effects (febrile neutropenia, neuropathy, fatigue, diarrhea) for each relevant reference arms from the studies included in this review.**
(DOCX)

Appendix S4 **Forest plot showing hazard ratio comparisons with 95% CI for PFS from meta-analyses of direct comparisons between various systemic regimens for advanced pancreatic cancer.**
(TIFF)

Appendix S5 **Hazard ratio comparisons of PFS from network meta-analysis.** Median values given with 95% credible regions. HR expressed as experimental vs. control.
(TIFF)

Appendix S6 **Probabilities that each treatment regimen is the best based on PFS.**
(TIFF)

Appendix S7 **Odds ratio comparisons of objective response rate.** Median values given with 95% credible regions. HR expressed as experimental vs. control.
(TIFF)

Appendix S8 **Probabilities that each treatment regimen is the best in terms of objective response rate.**
(TIFF)

Appendix S9 **Table comparing OS results from direct pairwise comparisons (HR with 95% CI) and network meta-analysis (HR with 95% CR) for various chemotherapy regimen comparisons.**
(TIFF)

Appendix S10 **Odds ratio comparisons of febrile neutropenia rates.** Median values given with 95% credible regions. HR expressed as experimental vs. control.
(TIFF)

Appendix S11 **Odds ratio comparisons of grade 3 or 4 neuropathy rates.** Median values given with 95% credible regions. HR expressed as experimental vs. control.
(TIFF)

Appendix S12 **Odds ratio comparisons of grade 3 or 4 fatigue rates.** Median values given with 95% credible regions. HR expressed as experimental vs. control.
(TIFF)

Appendix S13 **Odds ratio comparisons of grade 3 or 4 diarrhea rates.** Median values given with 95% credible regions. HR expressed as experimental vs. control.
(TIFF)

Appendix S14 **Risk of bias graph for all included trials.**
(TIFF)

Appendix S15 **Risk of bias summary for all included trials.**
(TIFF)

Checklist S1 **Completed PRISMA Checklist for reporting a systematic review and/or meta-analysis.**
(PDF)
