# Supplementary figures and images for: A Bayesian Meta-Analysis of Multiple Treatment Comparisons of Systemic Regimens for Advanced Pancreatic Cancer
Source: PLoS One. 2014 Oct 6;9(10):e108749. doi: 10.1371/journal.pone.0108749 (PMC4186762; doi:10.1371/journal.pone.0108749)

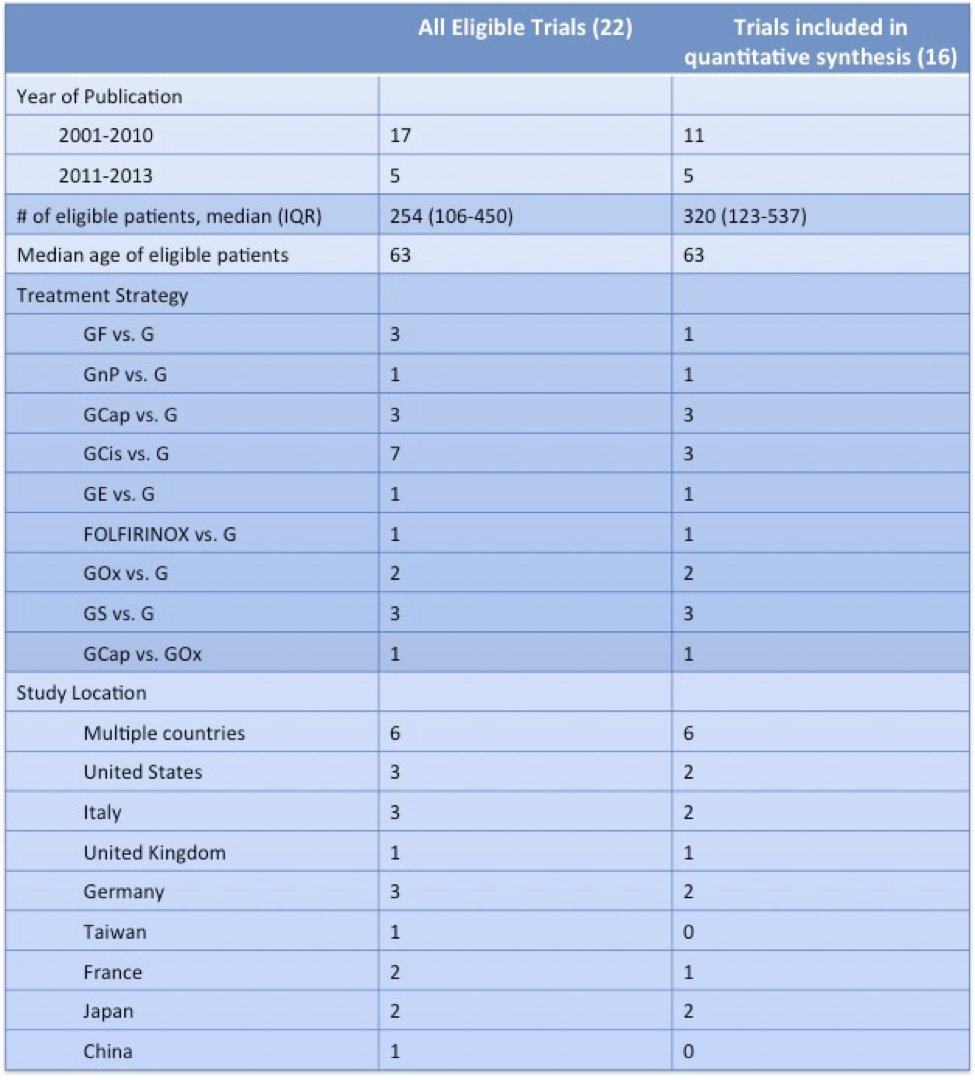

Supplement: Appendix S1 — Summary table of trial characteristics included in systematic review and quantitative synthesis. (TIFF) [file pone.0108749.s001.tiff]

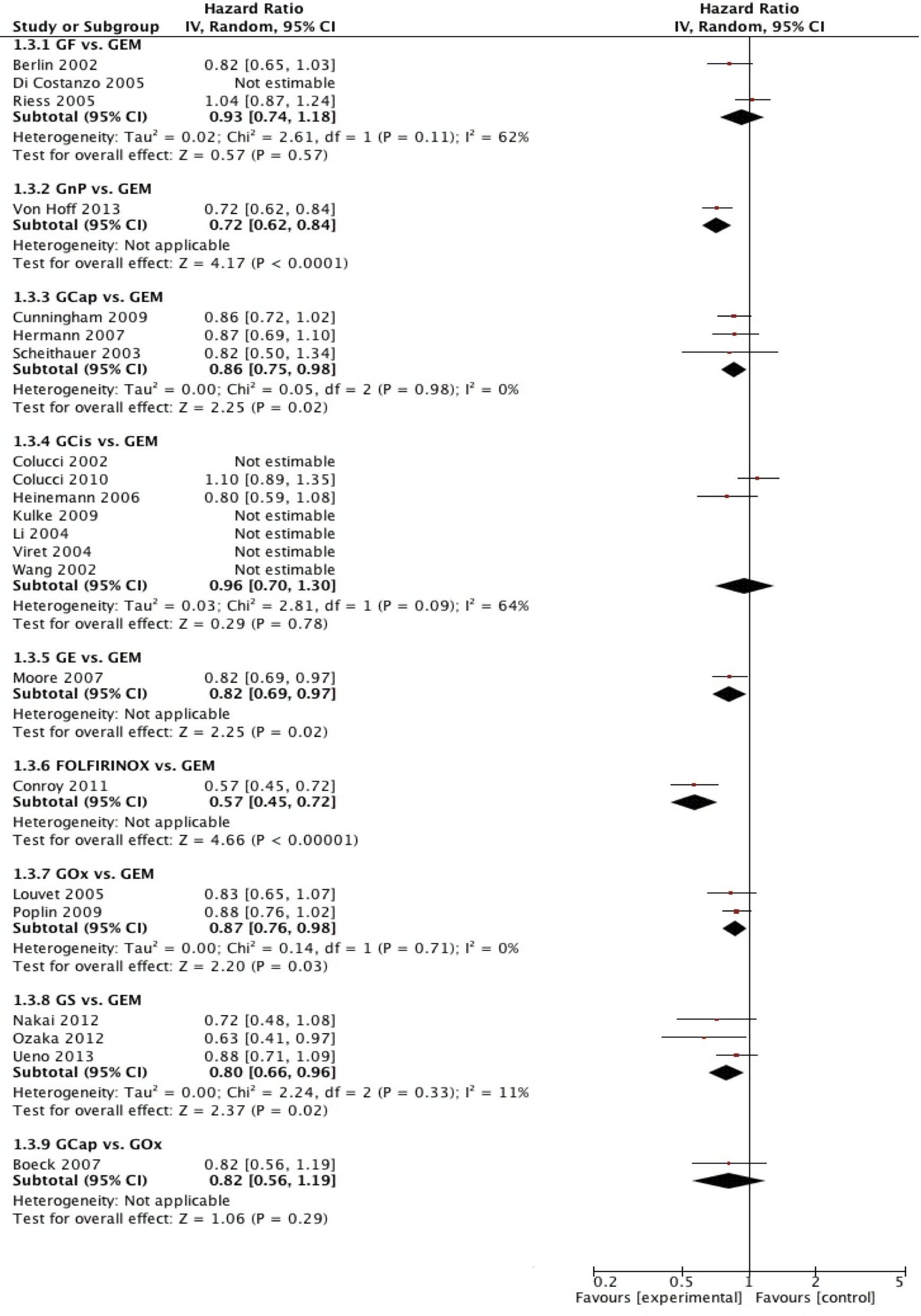

Supplement: Appendix S4 — Forest plot showing hazard ratio comparisons with 95% CI for PFS from meta-analyses of direct comparisons between various systemic regimens for advanced pancreatic cancer. (TIFF) [file pone.0108749.s004.tiff]

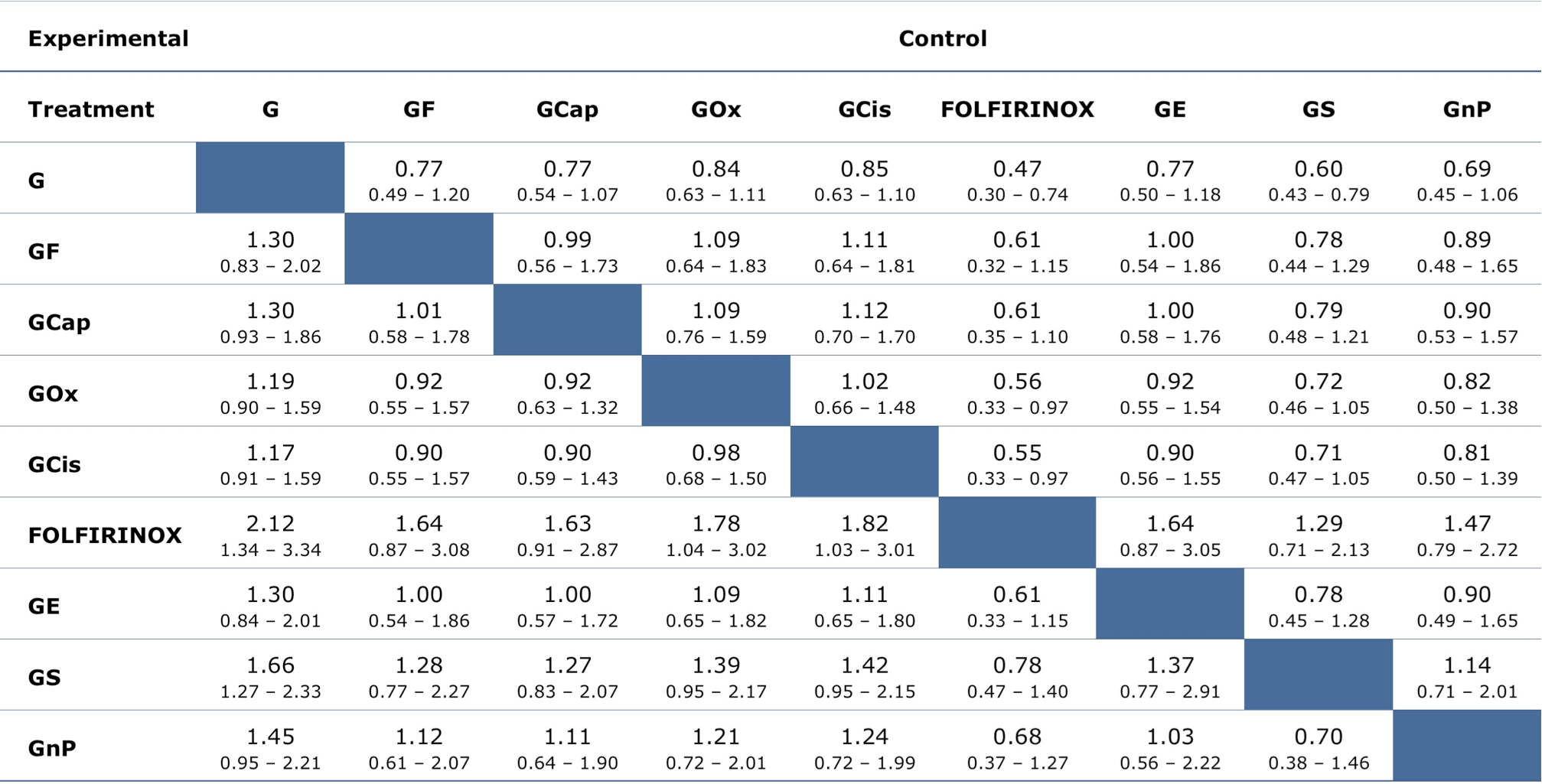

Supplement: Appendix S5 — Hazard ratio comparisons of PFS from network meta-analysis. Median values given with 95% credible regions. HR expressed as experimental vs. control. (TIFF) [file pone.0108749.s005.tiff]

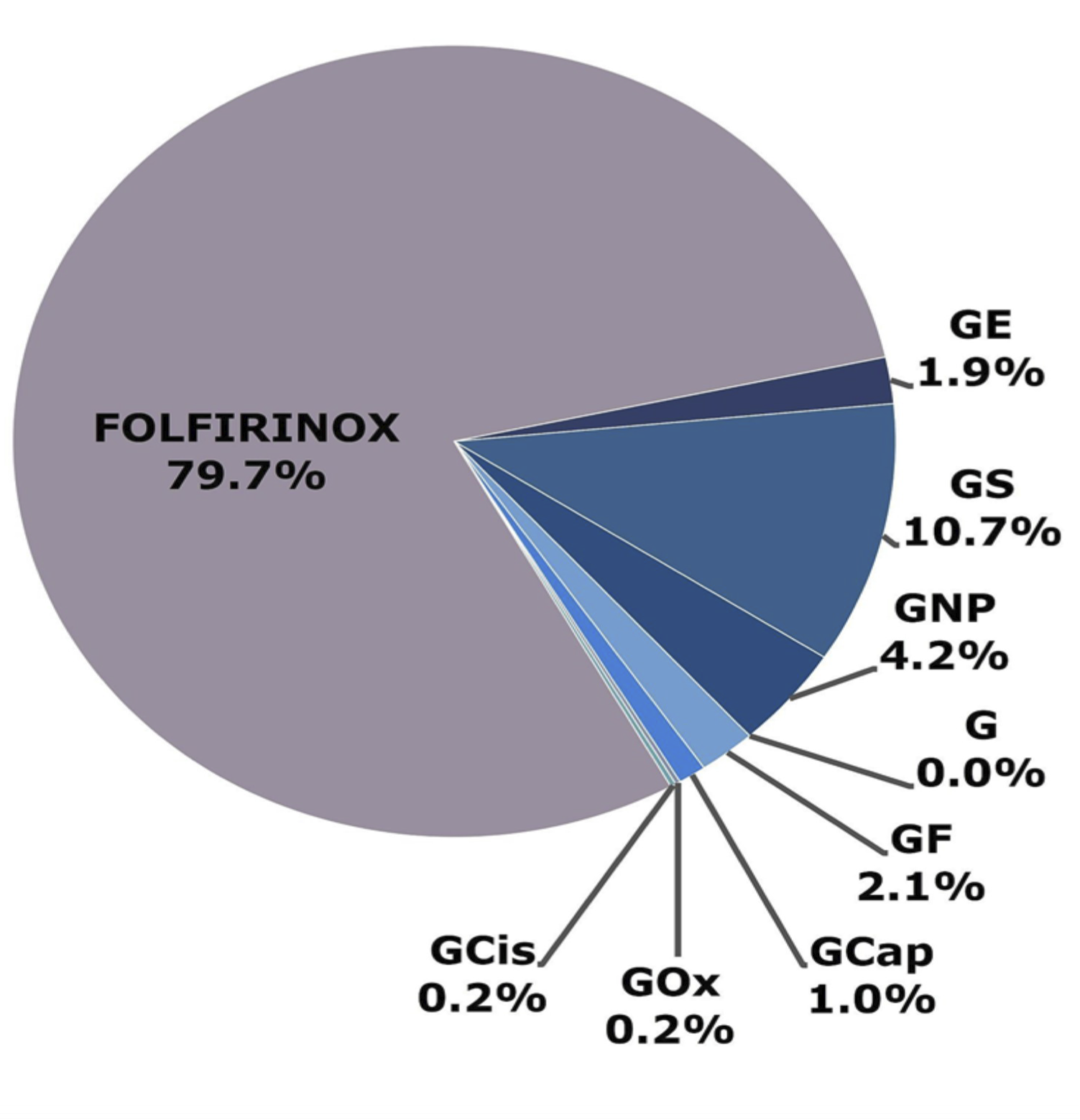

Supplement: Appendix S6 — Probabilities that each treatment regimen is the best based on PFS. (TIFF) [file pone.0108749.s006.tiff]

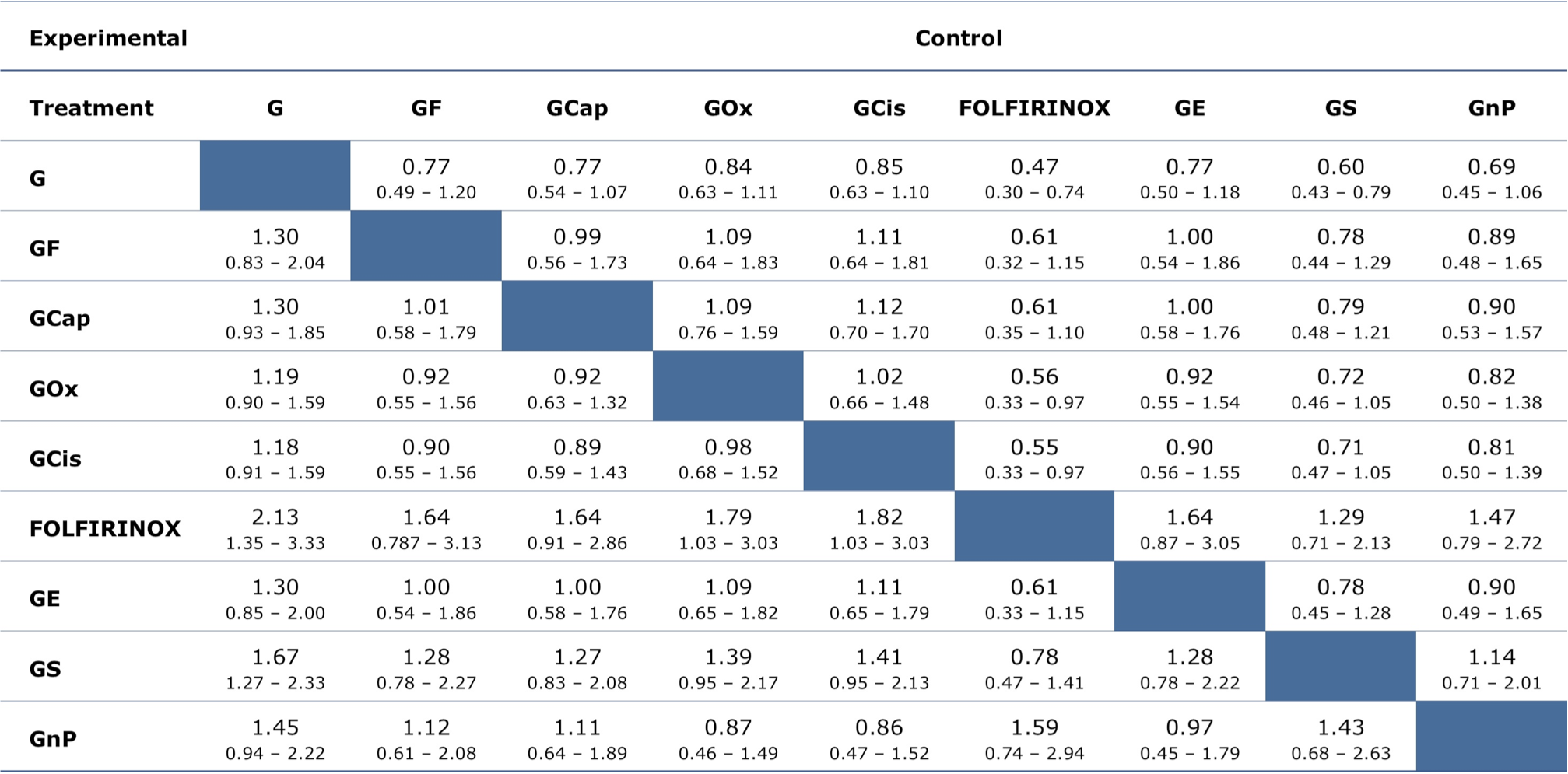

Supplement: Appendix S7 — Odds ratio comparisons of objective response rate. Median values given with 95% credible regions. HR expressed as experimental vs. control. (TIFF) [file pone.0108749.s007.tiff]

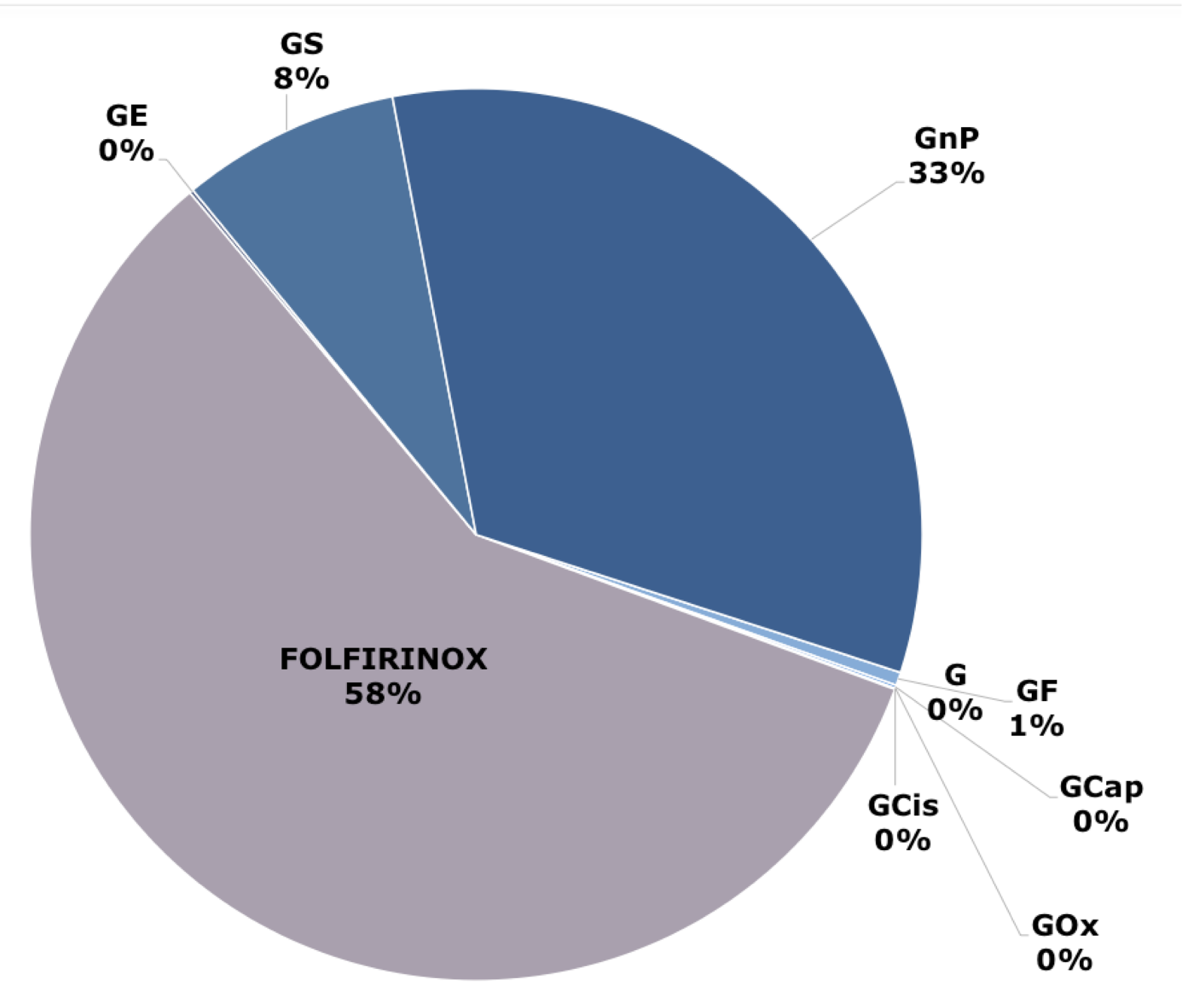

Supplement: Appendix S8 — Probabilities that each treatment regimen is the best in terms of objective response rate. (TIFF) [file pone.0108749.s008.tiff]

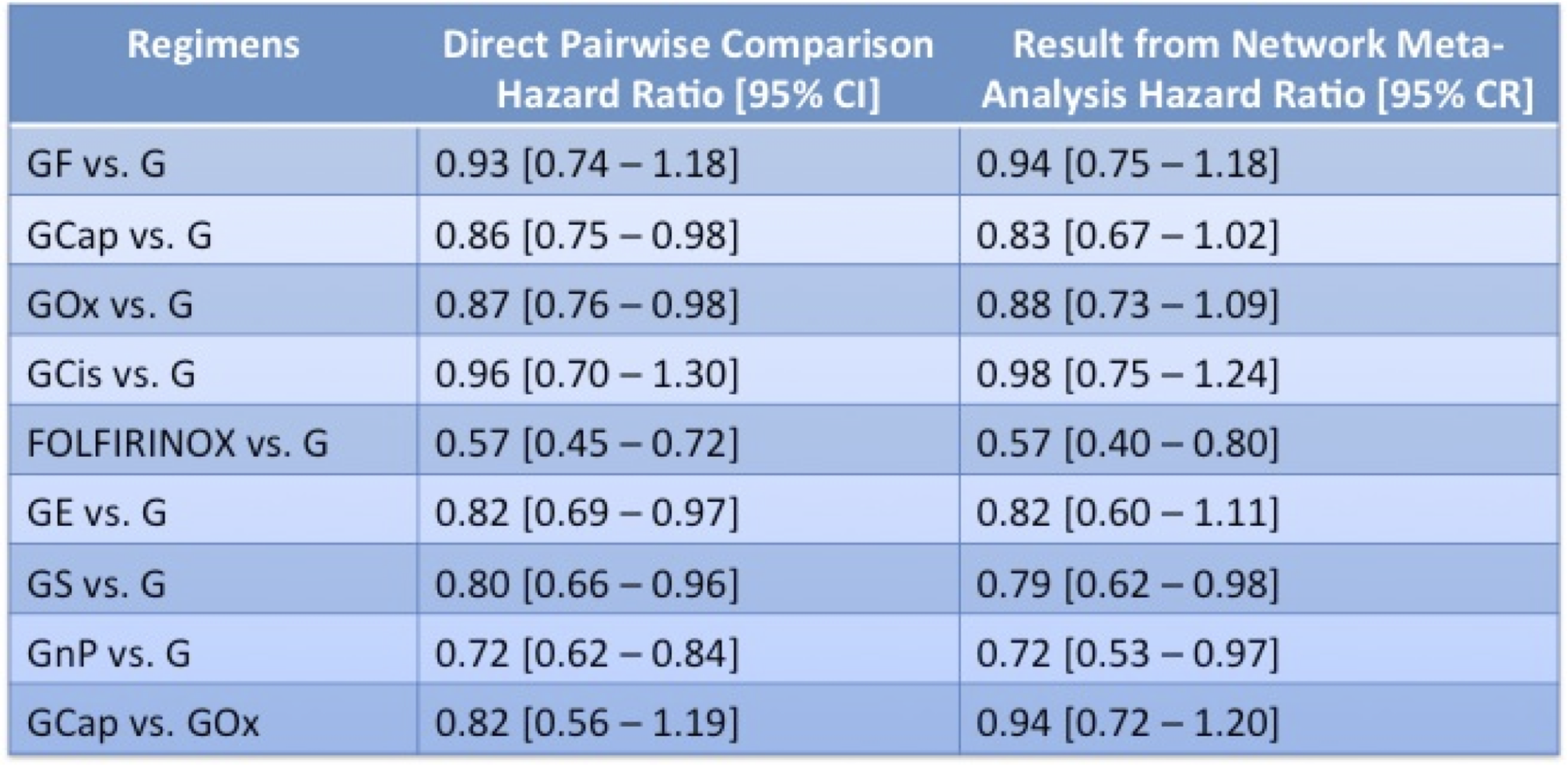

Supplement: Appendix S9 — Table comparing OS results from direct pairwise comparisons (HR with 95% CI) and network meta-analysis (HR with 95% CR) for various chemotherapy regimen comparisons. (TIFF) [file pone.0108749.s009.tiff]

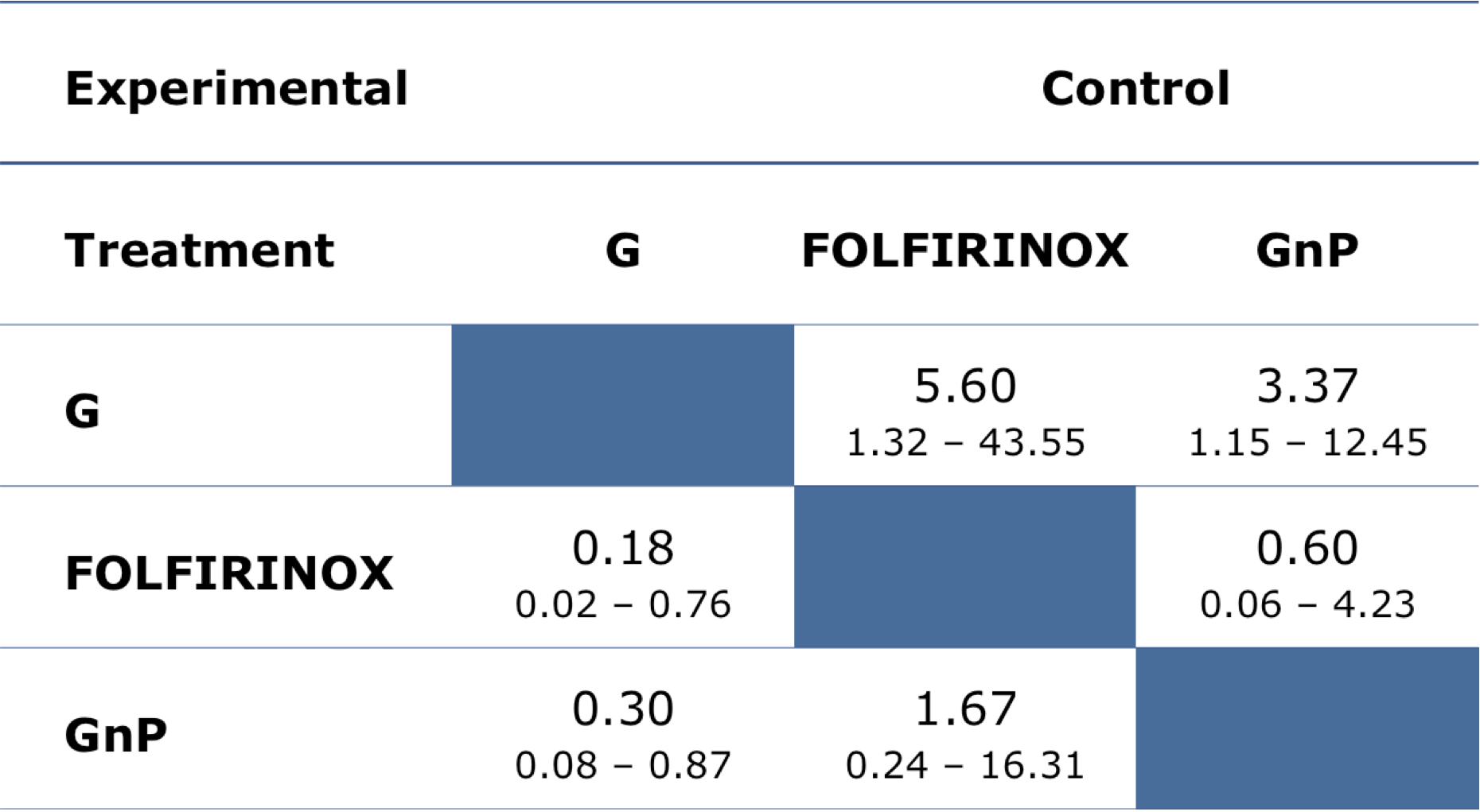

Supplement: Appendix S10 — Odds ratio comparisons of febrile neutropenia rates. Median values given with 95% credible regions. HR expressed as experimental vs. control. (TIFF) [file pone.0108749.s010.tiff]

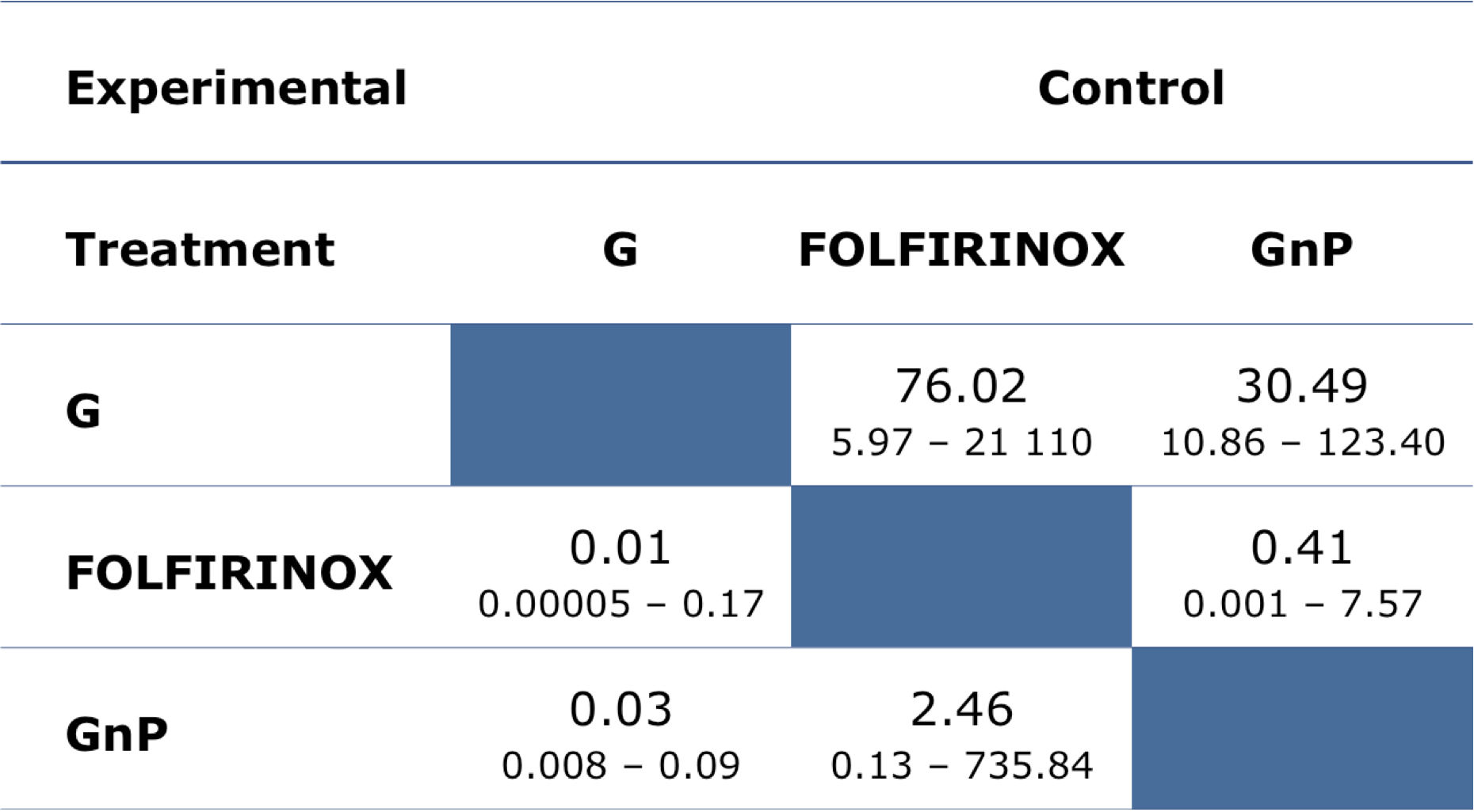

Supplement: Appendix S11 — Odds ratio comparisons of grade 3 or 4 neuropathy rates. Median values given with 95% credible regions. HR expressed as experimental vs. control. (TIFF) [file pone.0108749.s011.tiff]

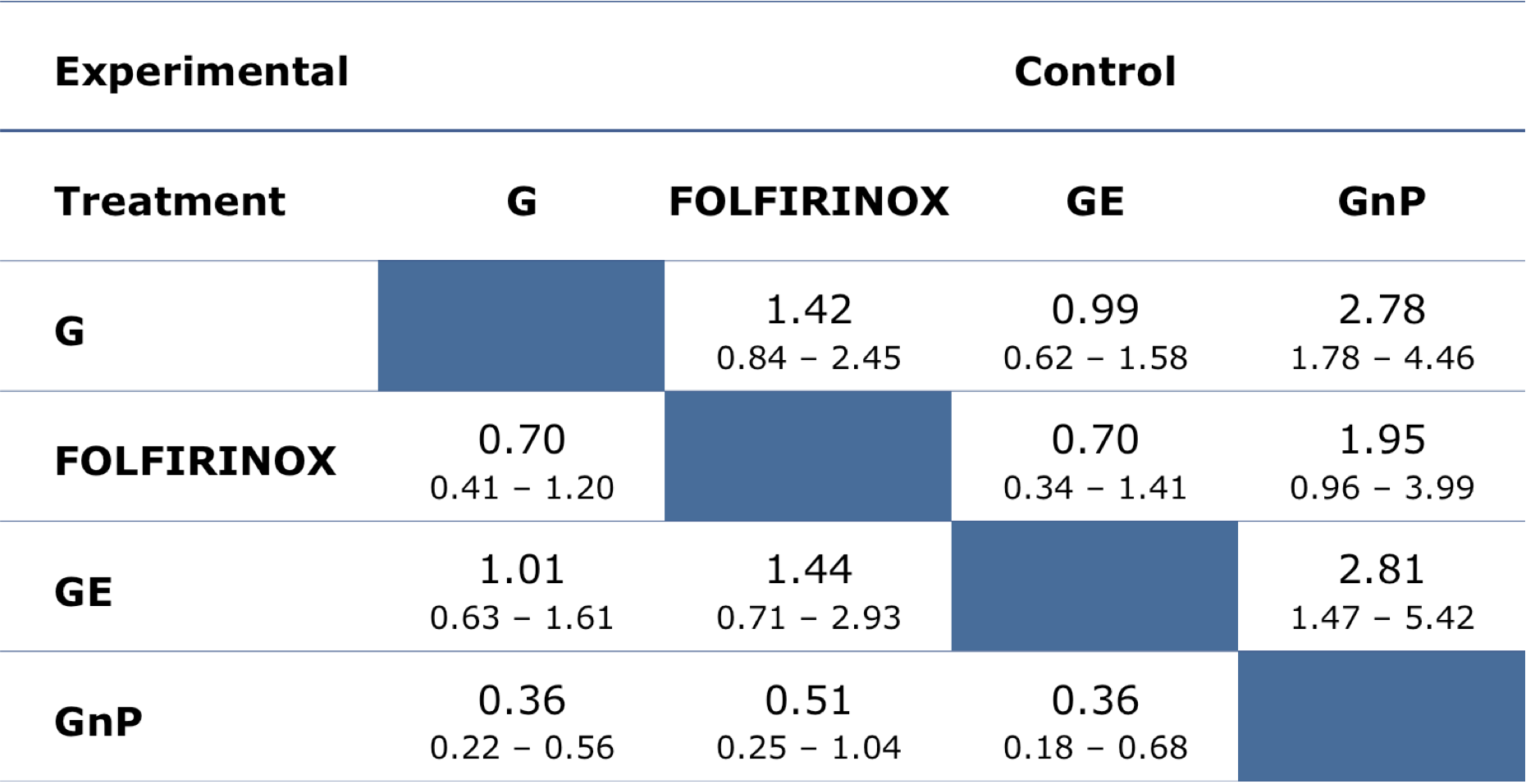

Supplement: Appendix S12 — Odds ratio comparisons of grade 3 or 4 fatigue rates. Median values given with 95% credible regions. HR expressed as experimental vs. control. (TIFF) [file pone.0108749.s012.tiff]

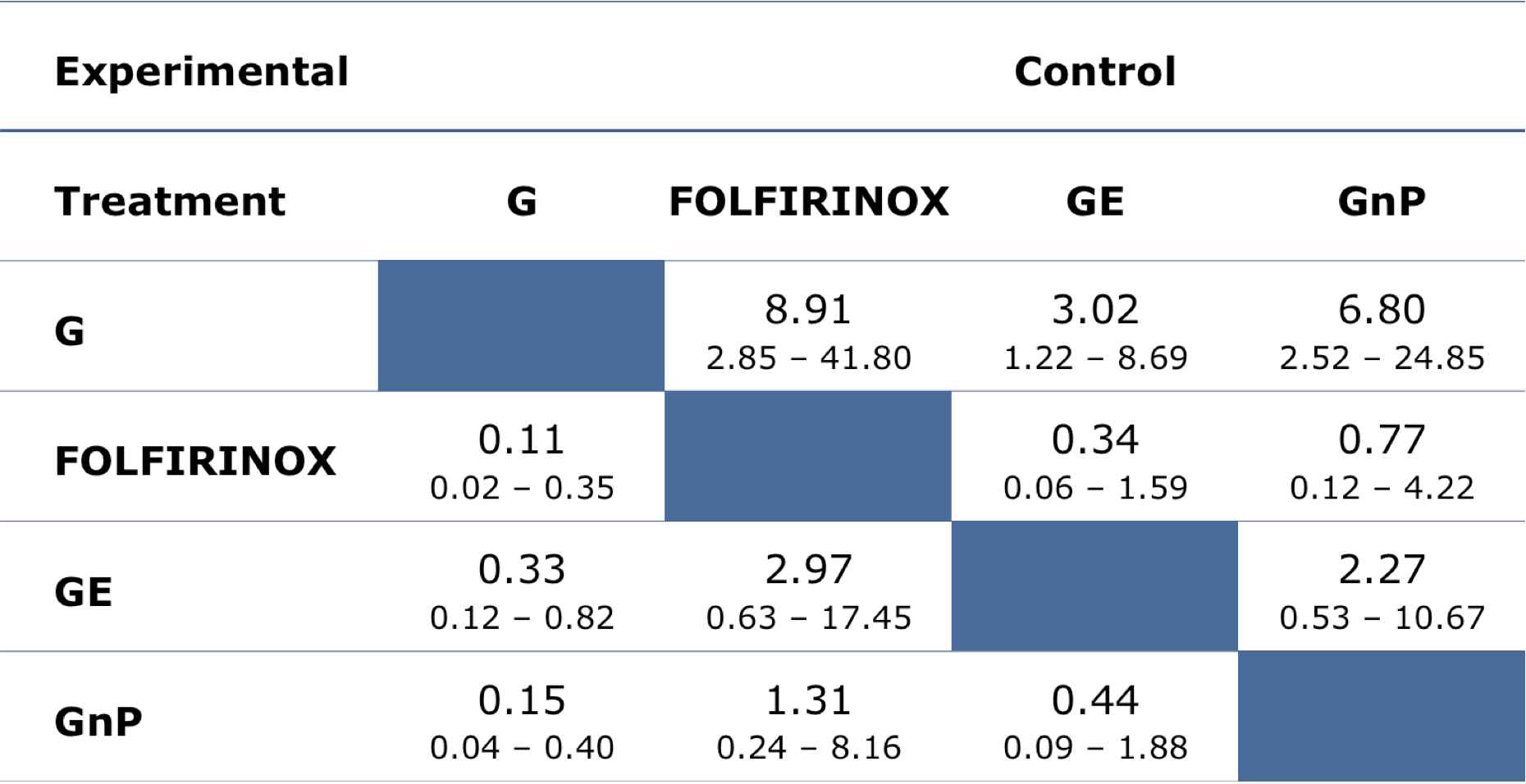

Supplement: Appendix S13 — Odds ratio comparisons of grade 3 or 4 diarrhea rates. Median values given with 95% credible regions. HR expressed as experimental vs. control. (TIFF) [file pone.0108749.s013.tiff]

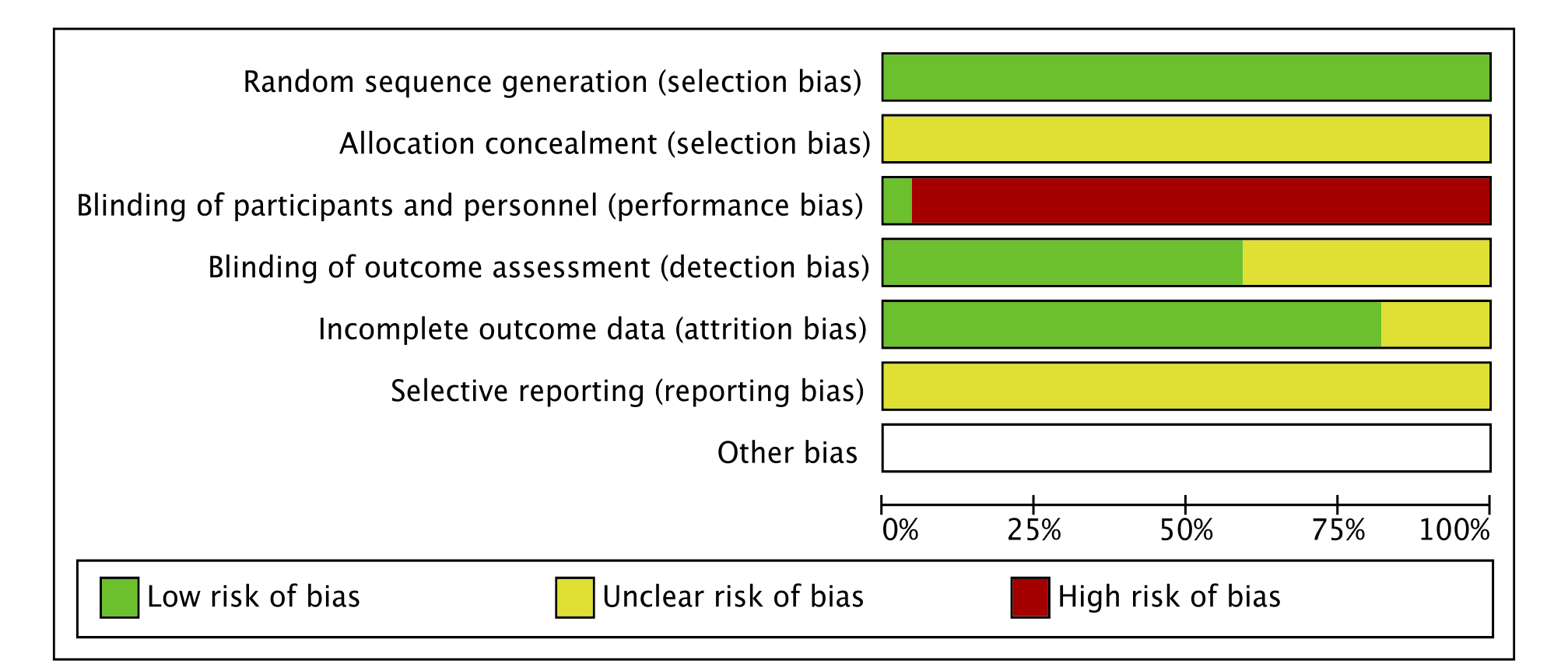

Supplement: Appendix S14 — Risk of bias graph for all included trials. (TIFF) [file pone.0108749.s014.tiff]

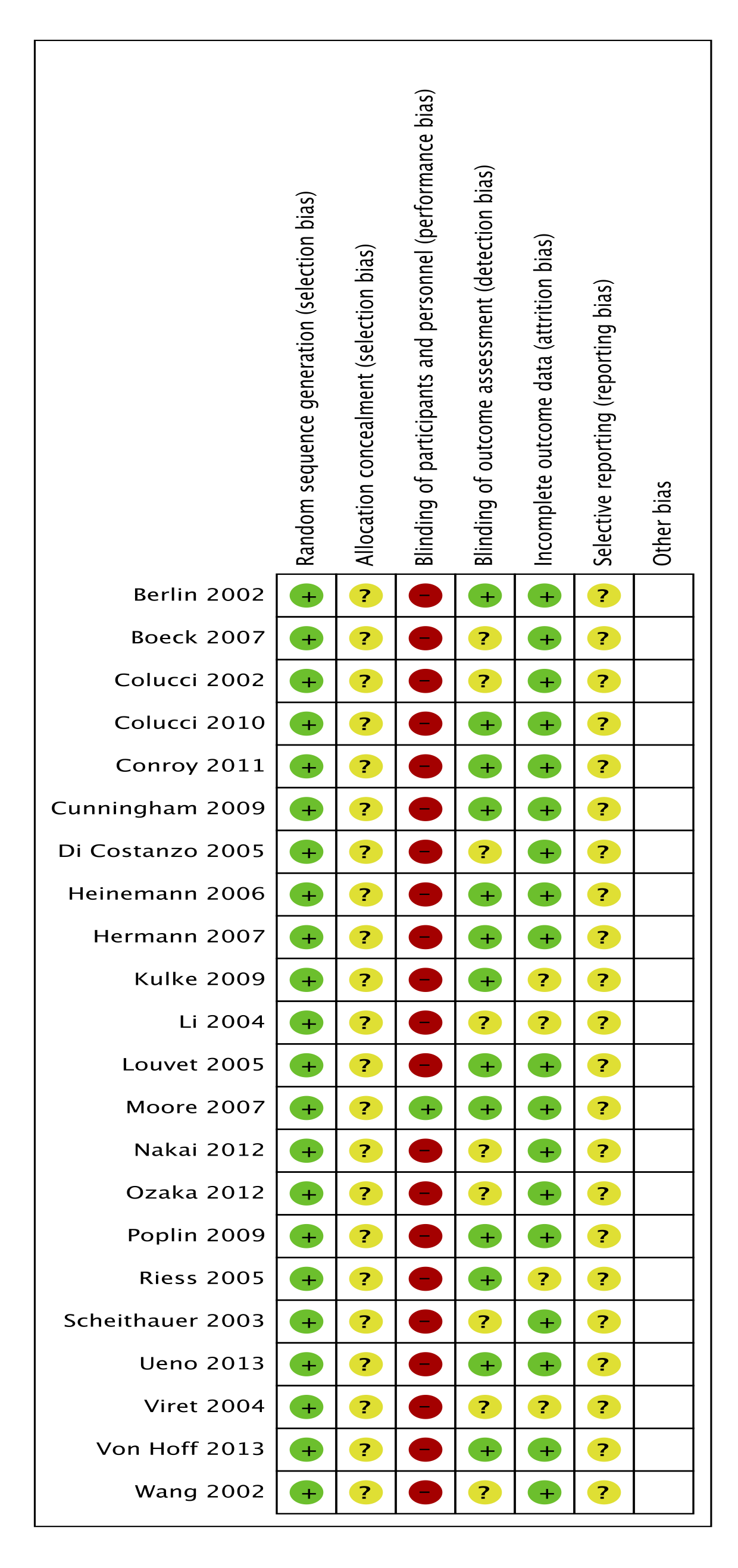

Supplement: Appendix S15 — Risk of bias summary for all included trials. (TIFF) [file pone.0108749.s015.tiff]
