# Supplementary material for: A Bayesian Meta-Analysis of Multiple Treatment Comparisons of Systemic Regimens for Advanced Pancreatic Cancer
Source: PLoS One. 2014 Oct 6;9(10):e108749. doi: 10.1371/journal.pone.0108749 (PMC4186762; doi:10.1371/journal.pone.0108749)
Supplement: Appendix S2 — Studies identified through the literature search. The geographic location of the institution of the primary investigator is described in the case where no study location was specified. (DOCX) [file pone.0108749.s002.docx]

Appendix II:

| **Study ID** | **Author** | **Year** | **Publication Type** | **Location** | **Outcomes** | **Arm** | **N** | **Intervention** | **Age (median)** | **Performance Status (%)** | **Stage** | |
| --- | --- | --- | --- | --- | --- | --- | --- | --- | --- | --- | --- | --- |
|  |  |  |  |  |  |  |  |  |  |  | **Locally Advanced** | **Metastatic** |
| 1 | Berlin | 2002 | Full manuscript | United  States | Primary: OS  Secondary: TTP, RR | GF | 160 | Gem 1000 mg/m^2^, bolus 5-FU 600 mg/m^2^ weekly; 3 weeks of 4 | 65.8 | ECOG 0: 22.8  ECOG 1: 63.6* P = 0.046  ECOG 2: 13.6 | 10.6 | 89.4 |
|  |  |  |  |  |  | G | 162 | Gem 1000 mg/m^2^ weekly; 3 weeks of 4 | 64.3 | ECOG 0: 34.6  ECOG 1: 51.8  ECOG 2: 13.6 | 9.9 | 90.1 |
| 2 | Boeck | 2007 | Full manuscript | Germany | Primary: PFS after 3 months  Secondary: OS, ORR, marker response, CBR, toxicity | GCap | 65 | Cap 825 mg/m^3^ po bid days 1-14, Gem 1000 mg/m^2^ day 1 and 8; every 3 weeks | 63 | KPS > 70: 86  KPS ≤ 70: 14 | 20 | 80 |
|  |  |  |  |  |  | GeGOx | 63 | Ox 130 mg/m^3^ day 8, Gem 1000 mg/m^2^ days 1 and 8; every 3 weeks | 63 | KPS > 70: 89  KPS ≤ 70: 11 | 16 | 84 |
| 3 | Colucci | 2002 | Full manuscript | Italy | Primary: TTP, CBR  Secondary: ORR, OS, toxicity | GCis | 53 | First cycle (7 weeks): CDDP 25 mg/m^2^ days 1, 8, 29, 36, and 42, Gem 1000 mg/m^2^ weekly; afterwards, 2 weeks rest  Following cycles: Both drugs weekly; 3 weeks of 4 | 60 | KPS median 70  Range: 50 - 100 | 40*missing 1 | 59* |
|  |  |  |  |  |  | G | 54 | First cycle: Gem 1000 mg/m^2^ weekly for 7 weeks; afterwards, 2 weeks rest  Following cycles: 3 weeks of 4 | 63 | KPS median: 70  Range: 50 - 100 | 50 | 50 |
| 4 | Colucci | 2010 | Full manuscript | Italy | Primary: OS  Secondary: PFS, ORR, toxicity, clinical benefit, QoL | GCis | 201 | First cycle (7 weeks): CDDP 25 mg/m^2^ days 1, 8, 29, 36, and 42, Gem 1000 mg/m^2^ weekly; afterwards, 1 week rest  Following cycles: Both drugs weekly; 3 weeks of 4 | 63 | KPS ≤70: 17.9  KPS ≥80: 82.1 | Stage 2, etc. 3.0, 12.4, 84.6 |  |
|  |  |  |  |  |  | G | 199 | First cycle: Gem 1000 mg/m^2^ weekly; afterwards, weeks rest  Following cycles: 3 weeks of 4 | 63 | KPS ≤70: 16.6  KPS ≥80: 83.4 | 4.5, 12.1, 82.9, missing 0.5 |  |
| 5 | Conroy | 2011 | Full manuscript | France | Primary: RR  Secondary: Safety | FOLFIRINOX | 171 | Ox 85 mg/m^2^ 2, leucovorin 400 mg/m^2^, irinotecan 180 mg/m^2^, fluorouracil 400 mg/m^2^ bolus then at 2400 mg/m^2^; every 2 weeks | 61 | ECOG 0: 37.4  ECOG 1: 61.9  ECOG 2: 0.6 | 0 | 100 |
|  |  |  |  |  |  | G | 171 | First cycle: Gem 1000 mg/m^2^ weekly; 7 weeks of 8  Following cycles: 3 weeks of 4 | 61 | ECOG 0: 38.6  ECOG 1: 61.4  ECOG 2: 0 | 0 | 100 |
| 6 | Cunningham | 2009 | Full manuscript | United Kingdom | Primary: OS  Secondary: PFS, ORR, toxicity, pain, QoL | GCap | 267 | Gem 1000 mg/m^2^, Cap 830 mg/m^2^ po bid weekly; 3 weeks of 4 | 62 | WHO 0: 25  WHO 1: 56  WHO 2: 19 | 30 | 70 |
|  |  |  |  |  |  | G | 266 | First cycle: Gem 1000 mg/m^2^ weekly; 7 weeks of 8  Following cycles: 3 weeks of 4 | 62 | WHO 0: 21  WHO 1: 61  WHO 2: 18 | 29 | 71 |
| 7 | Di Costanzo | 2005 | Full manuscript | Italy | Primary: RR  Secondary: OS, safety and tolerability | GF | 43 | First cycle (7 weeks): 5-FU 200 mg/m^2^ weekly, 6 weeks; Gem 1000 mg/m^2^ weekly; afterwards, 2 weeks rest  Following cycles: Both drugs; 3 weeks of 4 | 62 | KPS >80: 67  KPS <80: 33 | 33 | 67 |
|  |  |  |  |  |  | G | 48 | First cycle (7 weeks): Gem 1000 mg/m^2^ weekly; afterwards, 2 weeks rest  Following cycles: 3 weeks of 4 | 64 | KPS ≥80: 69  KPS <80: 31 | 27 | 73 |
| 8 | Heinemann | 2006 | Full manuscript | Germany | Primary: OS  Secondary: PFS, ORR, safety, QoL | GCis | 98 | Gem 1000 mg/m^2^, Cis 50 mg/m^2^ 1 hour weekly; 2 weeks of 4 | 64 | KPS 100: 25.3  KPS 90: 30.4  KPS 80: 34.2  KPS 70: 10.1 | 20 | 80 |
|  |  |  |  |  |  | G | 97 | Gem 1000 mg/m^2^ weekly; 3 weeks of 4 | 66 | KPS 100: 23.2  KPS 90: 25.6  KPS 80: 35.4  KPS 70: 15.8 | 21.1 | 78.9 |
| 9 | Hermann | 2007 | Full manuscript | Europe | Primary: OS  Secondary: PFS, ORR, safety QoL | GCap | 160 | Cap 650 mg/m^2^ po bid days 1-14, Gem 1000 mg/m^2^ days 1 and 8; every 3 weeks | NS | KPS 90 - 100: 54  KPS 60 - 80: 46 | 20 | 80 |
|  |  |  |  |  |  | G | 159 | First cycle: Gem 1000 mg/m^2^ weekly; 7 weeks of 8  Following cycles: 3 weeks of 4 | NS | KPS 90 - 100: 53  KPS 60 - 80: 47 | 21 | 79 |
| 10 | Kulke | 2009 | Full manuscript | United  States | Primary: OS  Secondary: toxicity, radiologic response, biochemical response, TTP | GCis | 66 | Gem 1000 mg/m^2^ days 1, 8 and 15, Cis 50 mg/m^2^ on days 1 and 15; every 4 weeks | 58.9 | ECOG 0: 24  ECOG 1: 65  ECOG 2: 11 | 0 | 100 |
|  |  |  |  |  |  | G | 64 | Gem 1500 mg/m^2^, rate of 10 mg/m^2^/min weekly; 3 weeks of 4 | 58.9 | ECOG 0: 24  ECOG 1: 62  ECOG 2: 14 | 0 | 100 |
| 11 | Li | 2004 | Abstract only | Taiwan | Primary: OS | GCis | 21 | Gem 1000 mg/m^2^, Cis 25 mg/m^2^ weekly; 3 weeks of 4 | NS | NS | 0 | 100 |
|  |  |  |  |  |  | G | 25 | Gem 1000 mg/m^2^ weekly; 3 weeks of 4 | NS | NS | 0 | 100 |
| 12 | Louvet | 2005 | Full manuscript | Europe | Primary: OS  Secondary: RR, CBR, PFS, safety | GOx | 157 | Gem 1000 mg/m^2^ day 1, Ox 100 mg/m^2^ day 2; every 2 weeks | Mean: 61.3 | WHO 0: 31  WHO 1: 52  WHO 2: 17 | 32 | 68 |
|  |  |  |  |  |  | G | 156 | First cycle: Gem 1000 mg/m^2^ weekly; 7 weeks of 8  Following cycles: 3 weeks of 4 | Mean: 60.1 | WHO 0: 28  WHO 1: 54  WHO 2: 30 | 30 | 70 |
| 13 | Moore | 2007 | Full manuscript | Multi-national | Primary: OS  Secondary: PFS, RR, response duration, toxicity, QoL, correlation of baseline tissue level with outcome | GE | 285 | First cycle: Gem 1000 mg/m^2^ weekly; 7 weeks of 8, Erl 100 or 150 mg/daily  Following cycles: Gem 3 weeks of 4, Erl 100 or 150 mg/daily | 63.7 | ECOG 0: 29.8  ECOG 1: 50.9  ECOG 2: 18.9 | 23.5 | 76.5 |
|  |  |  |  |  |  | G | 284 | First cycle: Gem 1000 mg/m^2^ weekly; 7 weeks of 8  Following cycles: 3 weeks of 4 | 64 | ECOG 0: 29.9  ECOG 1: 51.8  ECOG 2: 18.3 | 25 | 75 |
| 14 | Nakai | 2012 | Full manuscript | Japan | Primary: PFS  Secondary: OS, ORR, safety | GS | 53 | Gem 1000 mg/m^2^ days 1 and 8, S-1 bid; 4 weeks, then 2 weeks rest between cycles  Dose dependent on BSA (1.25 = 80 mg daily, 1.25 – 1.5 = 100 mg, >1.5 = 120 daily) | 63 | ECOG 0: 58.5  ECOG 1: 41.5  ECOG 2: 0 | 28.3 | 71.7 |
|  |  |  |  |  |  | G | 53 | Gem 1000 mg/m^2^ weekly; 3 weeks of 4 | 67 | ECOG 0: 60.4  ECOG 1: 37.7  ECOG 2: 1.9 | 24.5 | 75.5 |
| 15 | Ozaka | 2012 | Full manuscript | Japan | Primary; ORR  Secondary: toxicity, CBR, PFS, OS | GS | 53 | Gem 1000 mg/m^2^ days 1 and 8, S-1 40 mg/m^2^ bid; every 3 weeks | <65: 52.8  >65: 47.2 | ECOG 0: 83  ECOG 1 or 2: 17 | 24.5 | 75.5 |
|  |  |  |  |  |  | G | 59 | Gem 1000 mg/m^2^ weekly; 3 weeks of 4 | <65: 52.5  >65: 47.5 | ECOG 0: 76.3  ECOG 1 or 2: 23.7 | 30.5 | 69.5 |
| 16 | Poplin | 2009 | Full manuscript | United  States | Primary: OS  Secondary: toxicity, ORR, patterns of failure, PFS, symptom severity | GOx | 272 | Gem 1000 mg/m^2^ day 1, Ox 100 mg/m^2^ day 2; every 2 weeks | Mean: 63 | ECOG 0: 26.8  ECOG 1: 61.8  ECOG 2: 11 | 10.7 | 89.3 |
|  |  |  |  |  |  | G | 275 | First cycle: Gem 1000 mg/m^2^ weekly; 7 weeks of 8  Following cycles: 3 weeks of 4 | Mean: 63 | ECOG 0: 34.2  ECOG 1: 53.5  ECOG 2: 12.4 | 9.8 | 90.2 |
| 17 | Riess | 2005 | Abstract only | Germany | Primary: mOS  Secondary: TTP, toxicity | GF | 230 | Gem 1000 mg/m^2^, 5-FU 750 mg/m^2^, folinic acid 200 mg/m^2^ weekly; 4 weeks of 6 | 63 | KPS 60 - 80: 56 | stage Ivb: 76 |  |
|  |  |  |  |  |  | G | 236 | First cycle: Gem 1000 mg/m^2^ weekly; 7 weeks of 8  Following cycles: 3 weeks of 4 | 64 | KPS 60 - 80: 53 | stage Ivb: 77 |  |
| 18 | Scheithauer | 2003 | Full manuscript | Europe | Primary: PFS  Secondary: OS, RR | GCap | 41 | Gem 2200 mg/m^2^ diluted in 250 ml saline day 1, Cap 1250 mg/m^2^ po bid days 1-7; every 2 weeks | 64 | KPS 90 - 100: 27  KPS 70 - 80: 54  KPS 50 - 60: 19 | 0 | 100 |
|  |  |  |  |  |  | G | 42 | Gem 2200 mg/m^2^ diluted in 250 ml saline day 1; every 2 weeks | 66 | KPS 90 - 100: 24  KPS 70 - 80: 55  KPS 50 - 60: 21 | 0 | 100 |
| 19 | Ueno | 2013 | Full manuscript | Japan | Primary: OS  Secondary: PFS, RR | GS | 275 | Gem 1000mg/m^2^ days 1 and 8, S1 60, 80, or 100mg/d on day 1 through 14 of a 21 day cycle | NS | ECOG 0: 62.5  ECOG 1: 37.5 | 24.7 | 75.3 |
|  |  |  |  |  |  | G | 277 | Gem 1000 mg/m^2^ days 1, 8 and 15 | NS | ECOG 0: 65.3  ECOG 1: 34.7 | 23.8 | 76.2 |
| 20 | Viret | 2004 | Abstract only | France | Primary: TTF  Secondary: toxicity, OS, quality-adjusted time without symptoms or toxicity, QoL, duration of response | GCis | 42 | Gem 1000 mg/m^2^ days 1, 8 and 15, Cis 75 mg/m^2^ day 15; every 4 weeks | 61.5 | WHO PS 0 or 1: 76  WHO PS 2: 24 | stage III: 8 | stage IV: 34 |
|  |  |  |  |  |  | G | 41 | First cycle: Gem 1000 mg/m^2^ weekly; 7 weeks of 8  Following cycles: 3 weeks of 4 | 63 | WHO PS 0 or 1: 83  WHO PS 2: 17 | stage III: 9 | stage IV: 32 |
| 21 | Von Hoff | 2013 | Abstract and Full Manuscript | Multi-national | Primary: OS | GnP | 431 | *nab*-Paclitaxel 125 mg/m^2^, Gem 1000 mg/m^2^ weekly; 3 weeks of 4 | 62 | KPS 90 - 100: 58  KPS 70 - 80: 42 | 0 | 100 |
|  |  |  |  |  |  | G | 430 | First cycle: Gem 1000 mg/m^2^ weekly; 7 weeks  Following cycles: 3 weeks of 4 | 63 | KPS 90 - 100: 62  KPS 70 - 80: 38 | 0 | 100 |
| 22 | Wang | 2002 | Full manuscript (abstract in English) | China | Efficacy, CBR, toxicity | GCis | 16 | Gem 1000 mg/m^2^ days 1, 8 and 15, CDDP 60 mg/m^2^ day 15; every 4 weeks | NS | NS | NS | NS |
|  |  |  |  |  |  | G | 18 | First cycle: Gem 1000 mg/m^2^ weekly; 7 weeks of 8  Following cycles: 3 weeks of 4 | NS | NS | NS | NS |
