# Supplementary material for: A Bayesian Meta-Analysis of Multiple Treatment Comparisons of Systemic Regimens for Advanced Pancreatic Cancer
Source: PLoS One. 2014 Oct 6;9(10):e108749. doi: 10.1371/journal.pone.0108749 (PMC4186762; doi:10.1371/journal.pone.0108749)
Supplement: Appendix S3 — Extracted data for PFS, OS, ObRR, and side effects (febrile neutropenia, neuropathy, fatigue, diarrhea) for each relevant reference arms from the studies included in this review. (DOCX) [file pone.0108749.s003.docx]

| First Author | n | Reference Arm | Median PFS (months) | Median OS (months) | Objective Response Rate % | n | Febrile Neutropenia  (n) | Neuropathy  (n) | Fatigue  (n) | Diarrhea  (n) |
| --- | --- | --- | --- | --- | --- | --- | --- | --- | --- | --- |
| Berlin | 160 | 2 | 3.4 | 6.7 | 5.6 | 158 | - | 25 | - | 16 |
|  | 162 | 1 | 2.2 | 5.4 | 6.9 | 158 | - | 19 | - | 6 |
| Boeck | 64 | 3 | 5.7 | 9 | 25 | 63 | - | 0 | - | 3 |
|  | 63 | 4 | 3.9 | 6.9 | 13 | 60 | - | 5 | - | 5 |
| Colucci | 53 | 5 | 20 wks | 30 wks | 26.4 | 51 | - | - | - | 2 |
|  | 54 | 1 | 8 wks | 20 wks | 9.2 | 53 | - | - | - | 0 |
| Colucci | 201 | 5 | 3.8 | 7.2 | 12.9 | 186 | 0 | 2 | 10 | 1 |
|  | 199 | 1 | 3.9 | 8.3 | 10.1 | 189 | 1 | 0 | 6 | 3 |
| Conroy | 171 | 6 | 6.4 | 11 | 31.6 | 166 | 9 | 15 | 39 | 21 |
|  | 171 | 1 | 3.3 | 6.8 | 9.4 | 169 | 2 | 0 | 30 | 3 |
| Cunningham | 267 | 3 | 5.3 | 7.1 | 19.1 | 251 | - | - | - | 12 |
|  | 266 | 1 | 3.8 | 6.2 | 12.4 | 247 | - | - | - | 11 |
| diConstanzo | 43 | 2 | 18 wks | 30 wks | 11 | 41 | - | - | - | 0 |
|  | 48 | 1 | 14 wks | 31 wks | 8 | 49 | - | - | - | 0 |
| Heinemann | 98 | 5 | 5.3 | 7.5 | 11.5 | 90 | - | 0 | - | 3 |
|  | 97 | 1 | 3.1 | 6 | 9 | 85 | - | 0 | - | 4 |
| Hermann | 160 | 3 | 4.3 | 8.4 | - | 159 | 2 | - | - | 8 |
|  | 159 | 1 | 3.9 | 7.2 | - | 156 | 0 | - | - | 3 |
| Kulke | 66 | 5 | 4.5 | 6.7 | - | 62 | 0 | - | 10 | 0 |
|  | 64 | 1 | 3.3 | 6.4 | - | 58 | 0 | - | 8 | 1 |
| Li | 21 | 5 | 2.8 | 5.6 | 10 |  |  |  |  |  |
|  | 25 | 1 | 2.8 | 4.6 | 12 |  |  |  |  |  |
| Louvet | 157 | 4 | 5.8 | 9 | 26.8 | 157 | 2 | 30 | - | 9 |
|  | 156 | 1 | 3.7 | 7.1 | 17.3 | 156 | 2 | 0 | - | 2 |
| Moore | 285 | 7 | 3.75 | 6.24 | 8.6 | 282 | - | - | 42 | 17 |
|  | 284 | 1 | 3.55 | 5.91 | 8 | 280 | - | - | 42 | 5 |
| Nakai | 53 | 8 | 5.4 | 13.5 | 18.9 | 51 | 0 | - | 1 | 1 |
|  | 53 | 1 | 3.6 | 8.8 | 9.4 | 52 | 1 | - | 2 | 0 |
| Ozaka | 53 | 8 | 6.15 | 13.7 | 28.3 | 53 | - | - | 2 | 2 |
|  | 59 | 1 | 3.78 | 8 | 6.8 | 59 | - | - | 4 | 0 |
| Poplin | 272 | 4 | 2.7 | 5.7 | 9 | 263 | 1 | 66 | 45 | 16 |
|  | 275 | 1 | 2.6 | 4.9 | 6 | 264 | 1 | 0 | 50 | 9 |
| Riess | 230 | 2 |  | 5.85 |  |  |  |  |  |  |
|  | 236 | 1 |  | 6.2 |  |  |  |  |  |  |
| Scheithauer | 41 | 3 | 5.1 | 9.5 | 17 | 40 | - | - | 0 | 2 |
|  | 42 | 1 | 4 | 8.2 | 14 | 39 | - | - | 0 | 0 |
| Viret | 42 | 5 | 65 days | 241 days | 7 |  |  |  |  |  |
|  | 41 | 1 | 76 days | 201 days | 5 |  |  |  |  |  |
| Ueno | 275 | 8 | 5.7 | 10.1 | 29.3 | 267 | 5 | NS | 13 | 12 |
|  | 277 | 1 | 4.1 | 8.8 | 13.3 | 273 | 1 | NS | 10 | 3 |
| Von Hoff | 431 | 9 | 5.5 | 8.5 | 23 | 421 | 12 | 71 | 71 | 25 |
|  | 430 | 1 | 3.7 | 6.7 | 7 | 402 | 4 | 3 | 28 | 4 |
| Wang | 16 | 5 |  | 273 days | 31.3 |  |  |  |  |  |
|  | 18 | 1 |  | 217 days | 27.8 |  |  |  |  |  |
|  |  |  |  |  |  |  |  |  |  |  |
